# Impact of prior antihypertensive treatment on COVID-19 outcomes, by active ingredient

**DOI:** 10.1007/s10787-024-01475-2

**Published:** 2024-04-15

**Authors:** Rosa María García-Álvarez, Maruxa Zapata-Cachafeiro, Irene Visos-Varela, Almudena Rodríguez-Fernández, Samuel Pintos-Rodríguez, Maria Piñeiro-Lamas, Teresa M. Herdeiro, Adolfo Figueiras, Angel Salgado-Barreira, COVID-Drug Group, Rosendo Bugarín-González, Eduardo Carracedo-Martínez, Francisco J. González-Barcala, Martina Lema-Oreiro, Narmeen Mallah, Manuel Portela-Romero, Angela Prieto-Campo, Marc Saez, Margarita Taracido-Trunk

**Affiliations:** 1 Service of Preventive Medicine and Public Health, Clinic Hospital of Santiago de Compostela, Santiago de Compostela, Spain; 2 Department of Preventive Medicine and Public Health, University of Santiago de Compostela, 15786 Santiago de Compostela, Coruña, Spain; 3 Institute of Health Research of Santiago de Compostela, Santiago de Compostela, Spain; 4 Consortium for Biomedical Research in Epidemiology & Public Health (CIBER en Epidemiología y Salud Pública-CIBERESP), University of Santiago de Compostela, Santiago de Compostela, Spain; 5 Department of Medical Sciences, iBiMED-Institute of Biomedicine, University of Aveiro, Aveiro, Portugal

**Keywords:** Enalapril, Candesartan, Mortality, Hospitalization, COVID-19

## Abstract

**Objectives:**

To assess the impact of prior chronic treatment with angiotensin-converting enzyme inhibitors (ACEIs)/ angiotensin-receptor blockers (ARBs), both as a group and by active ingredient, on severity (risk of hospitalization and mortality), progression of and susceptibility to COVID-19.

**Methods:**

We conducted a multiple population-based case–control study in Galicia (north-west Spain). The study data were sourced from medical, administrative and clinical databases. We assessed: (1) risk of hospitalization, by selecting all patients hospitalized due to COVID-19 with PCR + as cases, and a random sample of subjects without a PCR + as controls; (2) COVID-19 mortality risk; (3) risk of disease progression; and (4) susceptibility to SARS-CoV-2, considering all patients with PCR + as cases, and the same subjects used in the previous model as controls. Adjusted odds ratios (aORs) were calculated.

**Results:**

ACEIs and ARBs were shown to decrease the risk of hospitalization (aOR = 0.78 [95%CI 0.69–0.89] and aOR = 0.80 [95%CI 0.72–0.90] respectively), risk of mortality (aOR = 0.71 [95%CI 0.52–0.98] and aOR = 0.69 [95%CI 0.52–0.91] respectively), and susceptibility to the virus (aOR = 0.88 [95%CI 0.82–0.94] and aOR = 0.92 [95%CI 0.86–0.97] respectively). By active ingredient: use of ***enalapril*** was associated with a significantly lower risk of hospitalization (aOR = 0.72 [95%CI 0.61–0.85]), mortality (aOR = 0.59 [95%CI 0.38–0.92]) and susceptibility to COVID-19 (aOR = 0.86 [95%CI 0.79–0.94]); and use of ***candesartan*** was associated with a decreased risk of hospitalization (aOR = 0.76 [95%CI 0.60–0.95]), mortality (aOR = 0.36 [95%CI 0.17–0.75]) and disease progression (aOR = 0.73 [95%CI 0.56–0.95]).

**Conclusion:**

This large-scale real-world data study suggest that ***enalapril*** and ***candesartan*** are associated with a considerable reduction in risk of severe COVID19 outcomes.

**Supplementary Information:**

The online version contains supplementary material available at 10.1007/s10787-024-01475-2.

## Introduction

The COVID-19 pandemic has highlighted the need to ascertain the effects of the use of different chronic medications on susceptibility to and severity of COVID-19. Identification of drugs that are associated with an increased risk would make it possible to opt for safer alternative treatments, whereas those associated with a decreased risk could be repurposed (WHO Solidarity Trial Consortium 2021) or be proposed as the most suitable option among medications sharing the same indication.

One of the therapeutic groups which has been and continues to be the focus of most debate is that of angiotensin-converting enzyme inhibitors (ACEIs) and angiotensin-receptor blockers (ARBs) (Danser et al. 2020; Fang et al. 2020; Wysocki et al. 2020), which are widely used for treatment of cardiovascular diseases such as hypertension and ischaemic heart disease López-Otero et al. 2021). This controversy arises because ACEIs/ARBs have several mechanisms whereby they can influence COVID-19 outcomes, in some cases with contrary effects: (i) on the one hand, they could increase the risk of COVID-19 outcomes, since in the medium term inhibition of ACE2 (angiotensin-converting enzyme 2) would bring about an increase in its receptors (Alhaddad et al. 2022; Asiimwe et al. 2022Udhaya et al., 2021), and this, on being the COVID-19 gateway, would in turn ***increase susceptibility*** to the virus (Gómez et al. 2020; Möhlendick et al. 2021). Furthermore: (ii) the increase in ACE2 would decrease the effects of angiotensin II involved in the renin–angiotensin–aldosterone system (RAAS), reducing the substrate for conversion of ACE2 into angiotensin 1–7 and so reducing its protective effect in the lung (Asiimwe et al. 2022), which ***would increase risk of progression*** to more severe stages of the disease. On the other hand: (iii) it has been seen that an increase in ACE2 (free or blocked by ACEIs/ARBs) could be associated with a decrease in the serum levels of inflammatory markers (Alhaddad et al. 2022), which would lead to a ***lower risk of progression*** to more severe stages among subjects who were COVID-positive (Baral et al. 2021; Kumar & Banerjee 2021; Meng et al. 2020).

A large number of observational studies have been published, which have been combined in several systematic reviews and meta-analyses (Aparisi et al. 2022; Baral et al. 2021; Caravaca et al. 2020; Kurdi et al. 2023). However, the majority of available observational studies display important inconsistencies and a critical risk of biases (Loader et al. 2022) due to (1) inadequate control of confounding biases; (2) selection bias; and (3) collider bias. Moreover, few of these studies are based on an uninfected population, thereby rendering them unable to assess the impact of these medications on susceptibility and risk of hospitalization. Likewise, available clinical trials evaluate the impact of medications on an already infected population (Asiimwe et al. 2022; Gnanenthiran et al. 2022), so that they too are unable to assess the impact of prior use and/or chronic use of these medications on susceptibility, a critical aspect in these medications, since one of the possible ACEI/ARB mechanisms of action implicated is linked to an increased concentration of ACE2 receptors in the lung (Asiimwe et al. 2022). Another important limitation of the studies available is the fact that few of them perform an analysis by active ingredient (Gnanenthiran et al. 2022), something which is highly relevant because it has been observed that there is not always a class effect in other pharmacological groups, and there may therefore be important differences by active ingredient (Visos-Varela et al. 2023).

Hence, the aim of our study was to assess the impact of prior chronic treatment with ACEIs/ARBs, by active ingredient, on severity (risk of hospitalization and mortality), disease progression and susceptibility to SARS-CoV-2**.** This would make it possible to identify the role of the different active ingredients in these groups on COVID-19 outcomes.

## Materials and methods

### Study design and participants

We conducted a population-based multiple case–control study (Rothman et al. 2008) in Galicia, targeting subjects over the age of 18 years covered by the Galician Health Service (GHS). This region has a population of 2.5 million patients, 98% of which is covered by GHS. The study period was March to December 2020.

### Cases and controls

Through exhaustive sampling, this design uses data on a representative sample of all cases. These data were compared against controls randomly drawn from the same population, thus providing a valid estimate of the prevalence of exposure and covariates in the population of origin (De Abajo et al. 2020).

**Fig 1:**
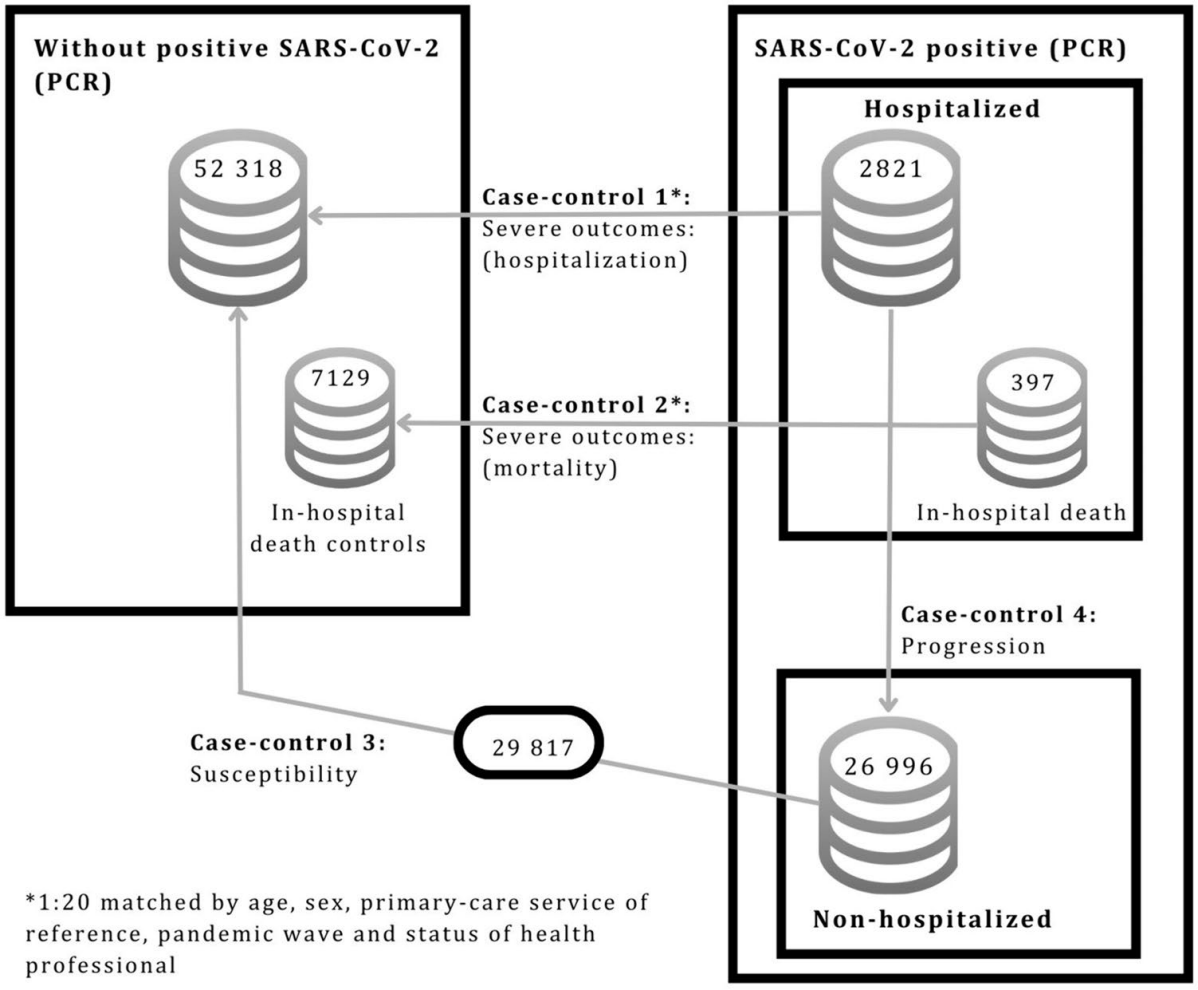
Population-based multiple case-control design

We conducted 4 case–control substudies which differed in their respective definitions of cases and controls in order to respond to each of the study objectives (Fig. 1 and Supplementary Table S1), namely, severity (hospitalization and mortality), susceptibility to the virus, and progression to severe COVID-19.

#### Case–control 1: severe COVID-19 outcomes – hospitalization

To assess the effect on risk of hospitalization, we defined cases as all subjects with diagnosis of COVID-19, confirmed by PCR test and hospitalised in a GHS hospital. We ruled out subjects hospitalized due to causes other than COVID-19, establishing for the purpose a maximum of 10 days’ difference between the date of the positive PCR test and that of hospitalization. Controls were selected by random sampling of the population that did not have a positive PCR test, and were matched by density of incidence, age, sex, primary care service of reference, and status of health professional, so as to ensure the same risk of exposure to SARS-CoV-2. Up to 20 controls per case were selected.

#### Case–control 2: severe COVID-19 outcomes – mortality

To assess the risk of mortality in patients with COVID-19, we defined cases as subjects with diagnosis of COVID-19, confirmed by a PCR test, who were hospitalized and died of COVID-19 during hospitalization at a GHS hospital. Controls were the subgroup of controls used in case–control substudy 1 (hospitalization) who were then matched with the cases of this substudy who had died during hospitalization.

#### Case–control 3: progression to severe COVID-19 outcomes

We assessed the effect of ACEIs/ARBs on progression to severe COVID-19 outcomes, and to this end used the same cases as case–control substudy 1 who were characterised by having required admission to a GHS hospital in Galicia. Controls were all patients with PCR-confirmed diagnosis of COVID-19 who did not require hospitalization.

Although these controls are not matched in this model, this does not affect the validity of the study, since the absence of matching does not cause biases but rather a decrease in study efficiency (Rose and van der Laan 2009; Rothman et al. 2008).

#### Case–control 4: susceptibility to the virus

We assessed risk of infection, defining cases as all subjects with a PCR-confirmed diagnosis of COVID-19 (both hospitalised and non-hospitalized). By way of a control group, we used the same controls as those of case–control substudy 1 who were characterised by the absence of a PCR-confirmed diagnosis of COVID-19. As in case–control substudy 3, the controls were not matched.

### Ethics committee

The study was approved by the Galician Clinical Research Ethics Committee (reference 2020–349), classified by the Spanish Medicines and Health Products Agency (*Agencia Española del Medicamentos y Productos Sanitarios/AEMPS*), and conducted in accordance with the principles of the Helsinki Declaration and the prevailing legislation governing biomedical research. The study protocol was registered in the electronic study registry following EU authorisation, EUPAS44587.

### Data-source and collection

All data were extracted automatically from the GHS Complex Data-Analysis Systems (*Sistemas de Información y Análisis Complejos/SIAC*) by an independent information technology (IT) services company (Visos-Varela et al. 2023).

As study covariates we collected demographic and anthropometric variables, clinical variables on COVID-19 (where applicable), data on hospitalisation, emergencies, deaths, comorbidities (arterial hypertension, diabetes mellitus, chronic obstructive pulmonary disease, obesity, cardiovascular disease, infection due to human immunodeficiency virus, chronic kidney failure, malignant neoplasm, and asthma), and exposure to all medications prescribed and dispensed to each of the subjects by retail pharmacies.

### Exposure

Exposure to the drug of interest was defined as current use, where an individual had a prescription issued and dispensed up to 6 months before the index date: in all other cases, exposure was defined as non-use. The index date was set as 10 days prior to the date of the PCR + test, or for non-PCR + tests, as the same index date as its matched case. We evaluated two sets of models: (1) grouped by ACEIs/ARBs; and (2) broken down by active ingredient.

### Statistical analysis

Risk of hospitalization, mortality, susceptibility to the virus, and progression to severe COVID-19 were assessed using multilevel logistic regression (Brown and Prescott 2015). These models were used because of the structure of the data and because they have many advantages over conditional regression (Brown & Prescott 2015; Pinheiro & Douglas, s. f.; Stroup 2012). Among other things, these advantages include the fact that: (1) they allow for analysis of matched and unmatched models; (2) they permit the inclusion of random terms to control for heterogeneity of initial clusters and time periods; and (3) strata in which cases match exposures with controls still count as events for calculation and for estimates.

We obtained adjusted odds ratios (aOR) for the effect of the ACEI/ARB treatment administered versus the lack of any treatment with ACEI/ARB medications. A subanalysis in hypertensive patients was performed with the same covariables as in the global analysis and the same analysis as at the global level. All analyses were performed using the free R Statistical Software environment (version 4.1.2). Statistical significance was set at 0.05.

Similarly, a subanalysis in hypertensive patients was performed for the 4 case–control substudies, responding to each of the study objectives (Supplementary Fig. 4).

## Results

The study covered a total of 82 135 subjects, comprising: 2821 cases (patients with a positive PCR test who required hospitalization), 397 of whom died during hospitalization; 26 996 non-hospitalized cases (patients with a positive PCR test who did not require hospitalization); and 52 318 patients without COVID-19 diagnosis during 2020 (Fig. 1). All the characteristics of the study subjects are shown in Tables 1 and 2.

**Table 1:** Demographic and clinical characteristics of COVID-19 cases and matched controls (severe outcomes: hospitalization and mortality)

	Severe COVID-19 outcomes			
	Hospitalization		Mortality	
Characteristic	CASES: PCR + hospitalized (N = 2821)	CONTROLS: non-PCR + (N = 52 318)	CASES: PCR + deceased (N = 397)	CONTROLS: non-PCR + (N = 7129)
Sex; n (%)				
Male	1457 (51.6)	26 998 (51.6)	236 (59.4)	4274 (60.0)
Female	1364 (48.4)	25 320 (48.4)	161 (40.6)	2855 (40.0)
Age, median (IQR)	74 (60 – 85)	73 (60 – 84)	84 (77 – 89)	84 (75 – 88)
Health professionals; n (%)	78 (2.8)	1203 (2.3)	0 (0.0)	0 (0.0)
Comorbidities; n (%)				
Hypertension	1639 (58.2)	26 292 (50.3)	295 (74.3)	4687 (65.7)
Diabetes	782 (27.8)	10 233 (19.6)	157 (39.5)	1760 (24.7)
COPD	369 (13.1)	4305 (8.2)	87 (21.9)	875 (12.3)
Obesity	830 (29.5)	10 104 (19.3)	114 (28.7)	1536 (21.5)
Ischaemic heart disease	326 (11.6)	4479 (8.6)	86 (21.7)	914 (12.8)
Cerebrovascular accident	277 (9.8)	3631 (6.9)	72 (18.1)	725 (10.2)
Heart failure	430 (15.3)	3780 (7.2)	106 (26.7)	796 (11.2)
Atrial fibrillation	425 (15.1)	5405 (10.3)	84 (21.2)	1137 (15.9)
Chronic renal failure	403 (14.3)	4059 (7.8)	99 (24.9)	882 (12.4)
Cancer	475 (16.9)	7277 (13.9)	98 (24.7)	1340 (18.8)
Asthma	267 (9.5)	3070 (5.9)	25 (6.3)	368 (5.2)
Current smoker	737 (26.1)	7842 (15.0)	83 (20.9)	866 (12.1)

*IQR* = interquartile range; *COPD* = chronic obstructive pulmonary disease

**Table 2:** Demographic and clinical characteristics of COVID-19 cases and matched controls (progression to severe COVID-19 outcomes and susceptibility to the virus)

	Progression to severe COVID-19 outcomes		Susceptibility to the virus	
Characteristic	CASES: PCR + hospitalized (N = 2821)	CONTROLS: PCR + non-hospitalized (N = 26 996)	CASES: PCR + hospitalized & non-hospitalized (N = 29 817)	CONTROLS: non-PCR + (N = 52 318)
Sex; n (%)				
Male	1457 (51.6)	11 217 (41.6)	12 674 (42.5)	26 998 (51.6)
Female	1364 (48.4)	15 779 (58.4)	17 143 (57.5)	25,320 (48.4)
Age, median (IQR)	74 (60 – 85)	47 (33 – 63)	49 (34 – 67)	73 (60 – 84)
Health professional; n (%)	78 (2.8)	1238 (4.6)	1316 (4.4)	1203 (2.3)
Comorbidities; n (%)				
Hypertension	1639 (58.2)	6208 (23.0)	7847 (26.3)	26 292 (50.3)
Diabetes	782 (27.8)	2519 (9.3)	3301 (11.1)	10 233 (19.6)
COPD	369 (13.1)	759 (2.8)	1128 (3.8)	4305 (8.2)
Obesity	830 (29.5)	3960 (14.7)	4790 (16.1)	10 104 (19.3)
Ischaemic heart disease	326 (11.6)	865 (3.2)	1191 (4.0)	4479 (8.6)
Cerebrovascular accident	277 (9.8)	867 (3.2)	1144 (3.8)	3631 (6.9)
Heart failure	430 (15.3)	678 (2.5)	1108 (3.7)	3780 (7.2)
Atrial fibrillation	425 (15.1)	1076 (4.0)	1501 (5.9)	5405 (10.3)
Chronic renal failure	403 (14.3)	712 (2.6)	1115 (3.7)	4059 (7.8)
Cancer	475 (16.9)	1755 (6.5)	2230 (7.5)	7277 (13.9)
Asthma	267 (9.5)	2170 (8.0)	2437 (8.2)	3070 (5.9)
Current smoker	737 (26.1)	4108 (15.2)	4845 (16.2)	7842 (15.0)

*IQR* = interquartile range; *COPD* = Chronic obstructive pulmonary disease

### Severe COVID-19 outcomes – hospitalization

Risk of hospitalization was assessed using 2821 cases and 52,318 controls (Fig. 1 and Suplementary Table S1). By pharmacological subgroup (Supplementary Fig. S1 and Tables S2 and S3), statistically significant differences were found in terms of a reduction in the risk of hospitalization for: ACEIs (aOR = 0.78, 95% [CI 0.69–0.89], p < 0.001), ARBs (aOR = 0.80, [95% CI 0.72–0.90], p < 0.001), and calcium-channel blockers (aOR = 0.83, [95% CI 0.73–0.95], p = 0.006).

A breakdown by active ingredient (Table 3 and 4 and Supplementary Figs. S2 and S3) showed that use of ***enalapril***, ***olmesartan***, ***valsartan***, ***candesartan*** and ***irbesartan*** was found to be associated with a statistically significant lower risk of hospitalization (aOR = 0.72, [95% CI 0.61–0.85], p < 0.001: aOR = 0.79, [95% CI 0.65–0.94], p = 0.010; aOR = 0.69, [95% CI 0.57–0.83], p < 0.001; aOR = 0.76, [95% CI 0.60–0.95], p = 0.018; aOR = 0.76, [95% CI 0.60–0.96], p = 0.022) respectively).

**Table 3:** Severe COVID-19 outcomes (ACEI): risk of hospitalization and mortality

	Severe COVID-19 outcomes							
	Risk of hospitalization				Risk of mortality			
	CASES: PCR + hospitalized (N = 2821)	CONTROLS: non-PCR + (N = 52 318)	Adjusted OR^a^ (95%CI)	P value	CASES: PCR + deceased (N = 397)	CONTROLS: non-PCR + (N = 7129)	Adjusted OR^a^ (95%CI)	P value
ACEIs (C09AA)	380 (13.5)^b^	6856 (13.1)^b^	0.78 (0.69–0.80)	< 0.001	61 (15.4)^b^	1120 (15.7)^b^	0.71 (0.52–0.98)	0.039
Captopril (C09AA01)	6 (0.2)	118 (0.2)	0.91 (0.40–2.09)	0.822	2 (0.5)	14 (0.2)	1.55 (0.32–7.51)	0.586
Enalapril (C09AA02)	183 (6.5)	3729 (7.1)	0.72 (0.61–0.85)	< 0.001	25 (6.3)	599 (8.4)	0.59 (0.38–0.92)	0.019
Lisinopril (C09AA03)	10 (0.4)	252 (0.5)	0.57 (0.30–1.08)	0.086	1 (0.3)	36 (0.5)	0.31 (0.04–2.37)	0.262
Ramipril (C09AA05)	142 (5)	2076 (4)	0.89 (0.73–1.07)	0.222	26 (6.5)	353 (5)	0.91 (0.58–1.42)	0.673
Quinapril (C09AA06)	4 (0.1)	75 (0.1)	0.99 (0.36–2.75)	0.989	1 (0.3)	11 (0.2)	1.20 (0.13–10.79)	0.868
Fosinopril (C09AA09)	1 (0)	28 (0.1)	0.68 (0.09–5.03)	0.703	1 (0.3)	6 (0.1)	2.56 (0.29–22.33)	0.395
Delapril (C09AA12)	4 (0.1)	103 (0.2)	0.59 (0.21–1.61)	0.303	1 (0.3)	15 (0.2)	0.84 (0.11–6.69)	0.871
Imidapril (C09AA16)	7 (0.2)	102 (0.2)	1.12 (0.51–2.43)	0.7812	2 (0.5)	17 (0.2)	1.80 (0.39–8.31)	0.454

*OR *odds ratio; *ACEIs *angiotensin converting enzyme inhibitors

^a^Adjusted for: sex, age, status of health professional, comorbidities (hypertension, diabetes, COPD, obesity, ischaemic heart disease, cerebrovascular accident, heart failure, atrial fibrillation, chronic renal failure, cancer, asthma, current smoker), current use of other pharmacological treatments and number of treatments for chronic diseases. Additionally, the primary-care service of reference and the pandemic wave were included as random effects

^b^The overall number of subjects exposed to ACEIs (C09AA) is lower than the sum of those exposed to the active ingredients of individual ACEIs (C09AA01, C09AA02, C09AA03, C09AA05, C09AA06, C09AA09, C09AA12, C09AA16), due to the fact that some subjects were exposed to more than one ACEI across the study period

**Table 4:** Severe COVID-19 outcomes (ARBs): risk of hospitalization and mortality

	Severe COVID-19 outcomes							
	Risk of hospitalization				Risk of mortality			
	CASES: PCR + hospitalized (N = 2821)	CONTROLS: non-PCR + (N = 52 318)	Adjusted OR^a^ (95%CI)	P-value	CASES: PCR + deceased (N = 397)	CONTROLS: non-PCR + (N = 7129)	Adjusted OR^a^ (95%CI)	P-value
ARBs (C09CA)	702 (24.9)^b^	12,427 (23.8)^b^	0.80 (0.72–0.90)	< 0.001	115 (29)^b^	2190 (30.7)^b^	0.69 (0.52–0.91)	0.008
Losartan (C09CA01)	158 (5.6)	2324 (4.4)	0.92 (0.77–1.10)	0.372	32 (8.1)	433 (6.1)	0.88 (0.58–1.34)	0.554
Eprosartan (C09CA02)	14 (0.5)	257 (0.5)	0.94 (0.54–1.62)	0.812	5 (1.3)	51 (0.7)	1.47 (0.56–3.84)	0.437
Valsartan (C09CA03)	139 (4.9)	2713 (5.2)	0.69 (0.57–0.83)	< 0.001	28 (7.1)	487 (6.8)	0.70 (0.45–1.09)	0.554
Irbesartan (C09CA04)	83 (2.9)	1649 (3.2)	0.76 (0.60–0.96)	0.022	12 (3)	291 (4.1º)	0.57 (0.30–1.05)	0.073
Candesartan (C09CA06)	89 (3.2)	1690 (3.2)	0.76 (0.60–0.95)	0.018	8 (2)	300 (4.2)	0.36 (0.17–0.75)	0.006
Telmisartan (C09CA07)	70 (2.5)	1078 (2.1)	0.97 (0.75–1.25)	0.800	6 (1.5)	182 (2.6)	0.54 (0.23–1.25)	0.149
Olmesartan (C09CA08)	159 (5.6)	2891 (5.5)	0.79 (0.65–0.94)	0.010	28 (7.1)	485 (6.8)	0.77 (0.49–1.20)	0.242

*OR* odds ratio; *ARBs *angiotensin II receptor blockers

^a^Adjusted for: sex, age, status of health professional, comorbidities (hypertension, diabetes, COPD, obesity, ischaemic heart disease, cerebrovascular accident, heart failure, atrial fibrillation, chronic renal failure, cancer, asthma, current smoker), current use of other pharmacological treatments and number of treatments for chronic diseases. Additionally, the primary-care service of reference and the pandemic wave were included as random effects

^b^The overall number of subjects exposed to ARBs (C09CA) is lower than the sum of those exposed to the active ingredients of individual ARBs (C09CA01, C09CA02, C09CA03, C09CA04, C09CA06, C09CA07, C09CA08), due to the fact that some subjects were exposed to more than one ARB across the study period

### Severe COVID-19 outcomes – mortality

Risk of hospitalization was assessed using 397 cases COVID-19 deaths and 7129 controls (Fig. 1, and Supplementary Table S1). Analysis by pharmacological subgroup showed that ACEIs and ARBs displayed significant differences in terms of a reduction in the risk of mortality (aOR = 0.71, [95% CI 0.52–0.98], p = 0.039; aOR = 0.69, [95% CI 0.52–0.91], p = 0.008 respectively) (Supplementary Tables S2 and S3). By active ingredient (Table 3 and 4 and Supplementary Figs. S2 and S3), however, statistically significant differences were found in terms of use leading to a decreased risk of mortality in patients with COVID-19 infection: ***enalapril*** (aOR = 0.59, [95% CI 0.38–0.92], p = 0.019) and ***candesartan*** (aOR = 0.36, [95% CI 0.17–0.75], p = 0.006).

### Progression to severe COVID-19 outcomes

Progression of PCR-positive COVID-19 subjects to greater severity possibly requiring hospitalisation was assessed on the basis of 2821 cases (positive PCR test, hospitalised) and 26 996 controls (cases with a positive PCR test, not hospitalised) (Fig. 1 and Supplementary Table 1). No statistically significant differences that might affect disease progression were found between the different pharmacological subgroups (Supplementary Tables S2 and S3).

By active ingredient (Tables 5 and 6 and Supplementary Figs. S2 and S3), only ***candesartan*** was shown to result in a statistically significant reduction in disease progression (aOR = 0.73, [95% CI 0.56–0.95], p = 0.022).

**Table 5:** Progression to severe COVID-19 outcomes and susceptibility to the virus (ACEI)

	Progression to severe COVID-19 outcomes				Susceptibility to the virus			
	CASES: PCR + cases hospitalized (N = 2821)	CONTROLS: PCR + non-hospitalized (N = 26 996)	Adjusted OR^a^ (95%CI)	P value	CASES: PCR + hospitalized & non-hospitalized (N = 29 817)	CONTROLS: non-PCR + (N = 52 318)	Adjusted OR^a^ (95%CI)	P value
ACEIs (C09AA)	380 (13.5)^b^	1513 (5.6)^b^	0.90 (0.77–1.06)	0.204	1893 (6.3)^b^	6856 (13.1)^b^	0.88 (0.82–0.94)	< 0.001
Captopril (C09AA01)	6 (0.2)	26 (0.1)	0.63 (0.23–1.74)	0.373	32 (0.1)	118 (0.2)	1.07 (0.70–1.63)	0.747
Enalapril (C09AA02)	183 (6.5)	811 (3)	0.84 (0.69–1.03)	0.087	994 (3.3)	3729 (7.1)	0.86 (0.79–0.94)	< 0.001
Lisinopril (C09AA03)	10 (0.4)	69 (0.3)	0.52 (0.25–1.05)	0.070	79 (0.3)	252 (0.5)	0.89 (0.67–1.17)	0.390
Ramipril (C09AA05)	142 (5)	455 (1.7)	1.08 (0.86–1.36)	0.526	597 (2)	2076 (4)	0.87 (0.79–0-97)	0.015
Quinapril (C09AA06)	4 (0.1)	13 (0)	1.57 (0.46–5.28)	0.470	17 (0.1)	75 (0.1)	0.93 (0.52–1.64)	0.798
Fosinopril (C09AA09)	1 (0)	4 (0)	0.81 (0.08–7.71)	0.853	5 (0)	28 (0.1)	0.77 (0.29–2.07)	0.604
Delapril (C09AA12)	4 (0.1)	13 (0)	1.07 (0.33–3.50)	0.905	17 (0.1)	103 (0.2)	0.62 (0.36–1.08)	0.092
Imidapril (C09AA16)	7 (0.2)	28 (0.1)	1.14 (0.44–2.95)	0.784	35 (0.1)	102 (0.2)	1.00 (0.66–1.53)	0.989

*OR* odds ratio; *ACEIs* angiotensin converting enzyme inhibitors

^a^Adjusted for: sex, age, status of health professional, comorbidities (hypertension, diabetes, COPD, obesity, ischaemic heart disease, cerebrovascular accident, heart failure, atrial fibrillation, chronic renal failure, cancer, asthma, current smoker), current use of other pharmacological treatments and number of treatments for chronic diseases. Additionally, the primary-care service of reference and the pandemic wave were included as random effects

^b^The overall number of subjects exposed to ACEIs (C09AA) is lower than the sum of those exposed to the active ingredients of individual ACEIs (C09AA01, C09AA02, C09AA03, C09AA05, C09AA06, C09AA09, C09AA12, C09AA16), due to the fact that some subjects were exposed to more than one ACEI across the study period

**Table 6:** Progression to severe COVID-19 outcomes and susceptibility to the virus (ARBs)

	Progression to severe COVID-19 outcomes				Susceptibility to the virus			
	CASES: PCR + cases hospitalized (N = 2821)	CONTROLS: PCR + non-hospitalized (N = 26 996)	Adjusted OR^a^ (95%CI)	P value	CASES: PCR + hospitalized & non-hospitalized (N = 29 817)	CONTROLS: non-PCR + (N = 52 318)	Adjusted OR^a^ (95%CI)	P value
ARBs (C09CA)	702 (24.9)^b^	2741 (10.2)^b^	0.88 (0.76–1.00)	0.057	3443 (11.5)^b^	12,427 (238)	0.92 (0.86–0.97)	0.005
Losartan (C09CA01)	158 (5.6)	535 (2)	1.00 (0.80–1.25)	0.986	693 (2.3)	2324 (4.4)	0.95 (0.86–1.06)	0.375
Eprosartan (C09CA02)	14 (0.5)	42 (0.2)	1.26 (0.64–2.50)	0.506	56 (0.2)	257 (0.5)	0.84 (0.62–1.14)	0.269
Valsartan (C09CA03)	139 (4.9)	654 (2.1)	0.80 (0.63–1.00)	0.051	703 (2.4)	2713 (5.2)	0.87 (0.79–0.96)	0.007
Irbesartan (C09CA04)	83 (2.9)	325 (1.2)	0.85 (0.64–1.13)	0.258	408 (1.4)	1649 (3.2)	0.82 (0.72–0.93)	0.002
Candesartan (C09CA06)	89 (3.2)	404 (1.5)	0.73 (0.56–0.95)	0.022	493 (1.7)	1690 (3.2)	0.98 (0.87–1.10)	0.682
Telmisartan (C09CA07)	70 (2.5)	199 (0.7)	1.16 (0.84–1.60)	0.361	269 (0.9)	1078 (2.1)	0.89 (0.77–1.03)	0.130
Olmesartan (C09CA08)	159 (5.6)	707 (2.6)	0.82 (0.66–1.02)	0.071	866 (2.9)	2891 (5.5)	0.97 (0.88–1.06)	0.507

*OR* odds ratio; *ARBs* angiotensin II receptor blockers

^a^Adjusted for: sex, age, status of health professional, comorbidities (hypertension, diabetes, COPD, obesity, ischaemic heart disease, cerebrovascular accident, heart failure, atrial fibrillation, chronic renal failure, cancer, asthma, current smoker), current use of other pharmacological treatments and number of treatments for chronic diseases. Additionally, the primary-care service of reference and the pandemic wave were included as random effects

^b^The overall number of subjects exposed to ARBs (C09CA) is lower than the sum of those exposed to the active ingredients of individual ARBs (C09CA01, C09CA02, C09CA03, C09CA04, C09CA06, C09CA07, C09CA08), due to the fact that some subjects were exposed to more than one ARB across the study period

### Susceptibility to the virus

The analysis covered a total of 82 135 subjects, 29 817 of whom were COVID-19 cases (patients with a positive PCR test, whether or not hospitalized) and 52 318 controls (subjects without diagnosis of COVID-19) (Fig. 1 and Supplementary Table 1). Overall, ACEIs showed significant differences in terms of a reduction in risk of susceptibility to COVID-19 (Supplementary Table S3) (aOR = 0.88, [95% CI 0.82–0.94], p < 0.001), as did ARBs (aOR = 0.92, [95% CI 0.86–0.97], p = 0.005).

By active ingredient (Table 5 and 6 and Supplementary Figs. S2 and S3), statistically significant differences were found in terms of a reduction in risk of susceptibility to the virus, for both ***enalapril*** (aOR = 0.86, [95% CI 0.79–0.94], p < 0.001) and ***ramipril*** (aOR = 0.87, [95% CI 0.79–0.97], p = 0.015). Similarly, within the ARB group, ***valsartan*** (aOR = 0.87, [95% CI 0.79–0.96, p = 0.007]) and ***irbesesartan*** (aOR = 0.82, [95% CI 0.72–0.93], p = 0.002) reduced susceptibility to COVID-19.

### Subnalysis of hypertensive patient

In the subnalysis of hypertensive patients, the analysis covered a total of 34 139 subjects, 7847 of whom were COVID-19 cases (patients with a positive PCR test, whether or not hospitalized) and 26 292 controls (subjects without diagnosis of COVID-19) (Supplementary Fig. 4 and Supplementary Tables 4, 5 and 6).

The results of the sensitivity analysis agree with the results of the global analysis (see Supplementary Tables 7–12 and Supplementary Figs. 5, 6 and 7). There were no major changes in the pharmacological subgroups and some small changes by active ingredient. Specifically for patients with arterial hypertension and COVID-19 infection, ***telmisartan*** shows reduction to virus susceptibility from aOR = 0.89 (95% CI 0.77–1.03) p = 0.130 to aOR = 0.86 (95% CI 0.73–1) p = 0.048 becoming significant with this subgroup analysis.

## Discussion

This large-scale real-world data (RWD) population-based case–control study has shown that ***enalapril*** and ***candesartan*** reduced the risk of severity of COVID-19 (lower risk of hospitalization and mortality). Given that these effects are not found for all ACEIs/ARBs, ***enalapril*** and ***candesartan***, could be the active ingredients to consider within these pharmacological groups in future COVID 19 emergency situations, and could even be candidate medications (Asiimwe et al. 2022) for use against other emerging viral diseases.

The results of our study indicate that, as a group, ACEIs/ARBs appear to be associated with a reduction in severity (mortality and hospitalization), something that would be consistent with the results of the most recent meta-analyses (Huang et al. 2023; Meng et al. 2020) performed for these groups of drugs. As in the case of other pharmacological groups (Visos-Varela et al. 2023), however, an appreciable degree of variability was detected in the effects depending upon the active ingredient, a finding that may prove highly relevant in clinical practice.

While a great number of studies have analysed COVID-19 outcomes by subgroups, very few have done so by active ingredient. Our results, as with studies on ***telmisartan, losartan, valsartan*** (Gnanenthiran et al. 2022) and ***ramipril*** (Ajmera et al. 2021; Asiimwe et al. 2022), showed no significant differences in terms of the effect of chronic use on mortality and hospitalization. Even so, ours is the first study to show that ***enalapril*** is associated with a decreased risk of hospitalization (aOR 0.72 [95% CI 0.61–0.85]), mortality (aOR 0.59 [95% CI 0.38–0.92]) and susceptibility (aOR 0.86 [95% CI 0.79–0.94]). We feel that there is little likelihood of this finding being due to chance or to some type of bias arising from the internal consistency between the results of the different outcomes.

In the case of ***candesartan***, our results show a lower risk of hospitalization (aOR 0.73 [95% CI 0.56–0.95]) and progression (aOR 0.73 [95% CI 0.56–0.95]), which would be in line with the clinical trial conducted by Lukito et al. (Lukito et al. 2021). In addition, our large sample size -something that is difficult to attain in clinical trials- enabled us to identify its association with a decrease in mortality (aOR 0.36 [95% CI 0.17–0.75]).

We feel that our findings are not only statistically significant, but also clinically relevant. Hence, the reductions in mortality of 41% (95%CI: 8%-62%) and 64% (95%CI: 25%-83%) associated with prior exposure to ***enalapril*** and ***candesartan*** respectively could indicate that these active ingredients might well be the ACEIs/ARBs of choice in a COVID-19 outbreak situation.

***Enalapril***, unlike other ACEIs, does not display adverse immunological effects that could, in part, account for the effects found in our study. Furthermore, ***enalapril*** has an anti-inflammatory effect, on blocking the degradation of bradykinin (vasodilator substance), which inhibits the inflammatory cascade (Pedrosa et al. 2021; Ridgway et al. 2022) associated with the harm caused by SARS-CoV-2 infection (Pedrosa et al. 2021). This mechanism could explain the appreciable decrease in risk of severity (hospitalisation and mortality) and susceptibility, as compared to other active ingredients in the same pharmacological subgroup.

The reduction in risk of severity and progression of the virus brought about by ***candesartan*** might be determined by: (i) its anti-inflammatory effects on the lung (Dasu et al. 2009; Pedrosa et al. 2021), thanks to the fact that it binds with high affinity to the AT1 receptor (Ridgway et al. 2022), and thus dissociates more slowly (Tamargo et al. 2006) and inhibits oedema and cytokine release; and (ii) its in vitro antiviral effect (Elkahloun and Saavedra 2020), due to its chemical structure (*bisphenyl tetrazoles* (Liu et al. 2006; Ridgway et al. 2022)).

Our results for ***enalapril*** and ***candesartan***, along with their mechanisms of action, suggest that, among ACEIs and ARBs, these two active ingredients could be drugs of choice in the face of new SARS-CoV-2 pandemics or outbreaks and could also play a similar role in the face of threats by other emerging viral infections, due to:

- their high effect magnitude observed in our data for the various outcomes;
- their safety and efficacy profile being similar to that of other active ingredients in the group;
- their low cost, a factor that might be especially important for low-and middle-income countries in which access to vaccines and antivirals is difficult; and,
- their potential effect on viral diseases with an important inflammatory component, e.g., influenza, zika (Loe et al. 2019), ebola, pneumonia (Fedson 2016) and dengue (Hernández-Fonseca et al. 2015), something that would suggest the need for more studies to be conducted into their potential effect on such diseases.

Our study design has a number of **strengths**: (1) in a region of approximately 3 million inhabitants it included all cases with positive diagnosis of COVID-19 in 2020, thus eliminating the possibility of selection bias; (2) for the first time, it made it possible to assess the effect of ambulatory use of antihypertensives on the entire natural history of COVID-19, ranging from susceptibility, through progression and hospitalization, to mortality; (3) our large sample size enabled us to assess the effects of each active ingredient, a key factor, since our initial hypothesis postulated that each active ingredient could display different effects; (4) our study allowed us to adjust for many confounding variables, such as socio-demographic factors, comorbidities, and use of other medications; (5) exposure was measured on the basis of administrative databases, something that reduces the risk of misclassification, though there may be a residual effect due to incomplete adherence to the treatment (Lam and Fresco 2015); (6) the models used and our results proved to be very robust, since the subanalysis of hypertensive patients (Patel and Verma 2020) showed very slight or negligible variations compared to the overall results. In view of our findings for ***enalapril*** and ***candesartan***, however, we feel that if there had been a lack of therapeutic adherence, this would underestimate the associations, which could, in turn, indicate that the beneficial effect might be even greater.

Important limitations must also be considered when interpreting the results of our study. Firstly, by virtue of it being an observational study with secondary databases, one cannot rule out that there may be variables which acted as confounding factors that were not measured or may have been misclassified. In the variables that were indeed collected (e.g., indication and pathology), the level of severity was not available to us, and there could thus be a risk of a certain degree of residual confounding. Secondly, the lack of matching in the susceptibility and progression substudies could be perceived as a limitation. Yet, according to Rose and Rothman, (Rose & Laan 2009; Rothman et al. 2008), lack of matching in case–control studies only reduces efficacy but has no influence on risk of bias. Thirdly, the data used pertain to 2020, a time when the alpha variant was predominant, and our results should thus be extrapolated with caution for any other type of variant. Fourthly, during the first months of the pandemic, there was a limited availability of diagnostic tests, which might possibly have resulted in some COVID-19 non-PCR + subjects in realty being asymptomatic COVID-19 subjects. Finally, one might think that the results obtained from ACEIs/ARBs on in-hospital mortality could be affected by in-hospital treatment. However, we have no reason to think that the in-hospital treatment received depends on which type of ACEIs/ARBs they take. Furthermore, patients prescribed an ACEIs/ARBs would be expected to have a higher cardiovascular risk, associated with worse COVID-19 outcomes, but despite this, the results suggest that these drugs decrease the risk.

In conclusion, the COVID-19 pandemic has led us to reflect on the need to use drug-repurposing as a strategy to combat global public health threats. The results of this large-scale RWD study suggest that ***enalapril*** and ***candesartan*** are associated with a sizeable reduction in risk of severe COVID19 outcomes. If these results were repeated with other databases and replicated in clinical trials, we feel that, given the magnitude of the effects found, this finding could well be relevant for preventing the impact of COVID-19. Moreover, our results, along with those of in vivo and in vitro studies, suggest the need for more research to evaluate these drugs’ potential effect against viral diseases with a major inflammatory component, present or future.

### Supplementary Information

Below is the link to the electronic supplementary material.

Supplementary file1 (TIF 1673 KB)

Supplementary file2 (TIF 1573 KB)

Supplementary file3 (TIF 1575 KB)

Supplementary file4 (TIF 630 KB)

Supplementary file5 (TIF 852 KB)

Supplementary file6 (TIF 573 KB)

Supplementary file7 (TIF 883 KB)

Supplementary file8 (DOCX 225 KB)

## Data Availability

Research data are not shared.
