# Supplementary figures and images for: Impact of prior antihypertensive treatment on COVID-19 outcomes, by active ingredient
Source: Inflammopharmacology. 2024 Apr 15;32(3):1805–15. doi: 10.1007/s10787-024-01475-2 (PMC11136854; doi:10.1007/s10787-024-01475-2)

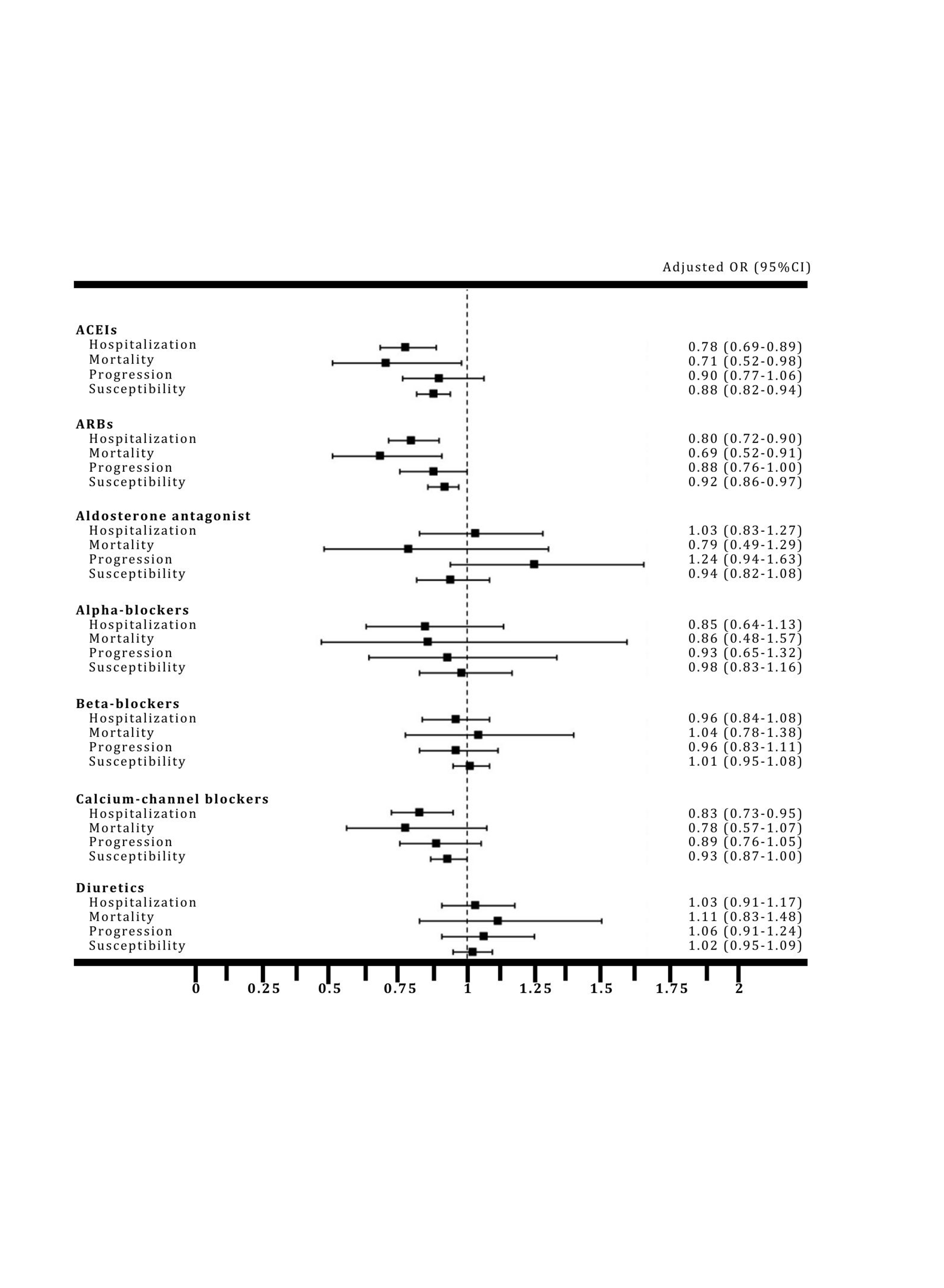

Supplement: Supplementary file 1 — Supplementary file1 (TIF 1673 KB) [file 10787_2024_1475_MOESM1_ESM.tif]

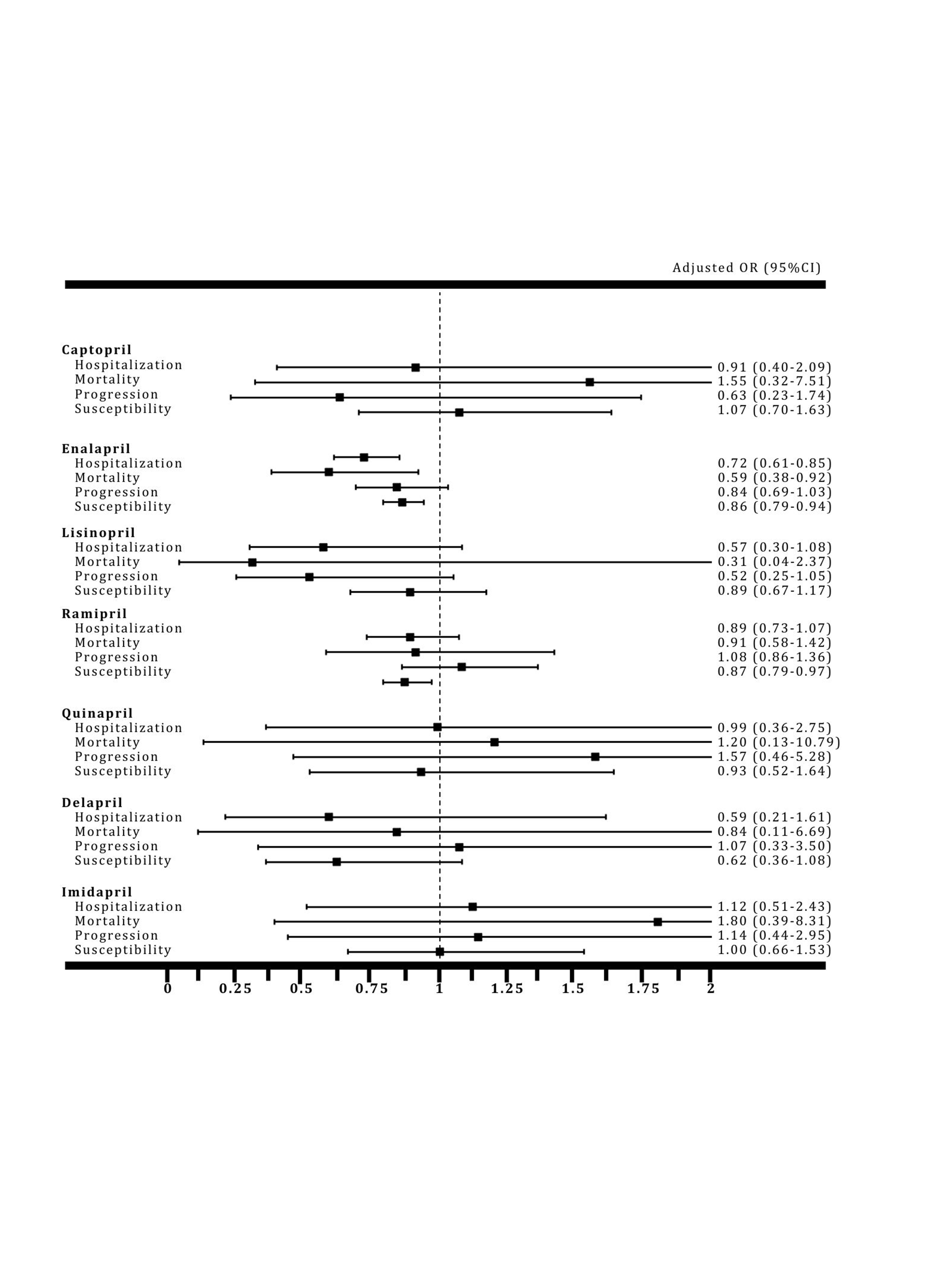

Supplement: Supplementary file 2 — Supplementary file2 (TIF 1573 KB) [file 10787_2024_1475_MOESM2_ESM.tif]

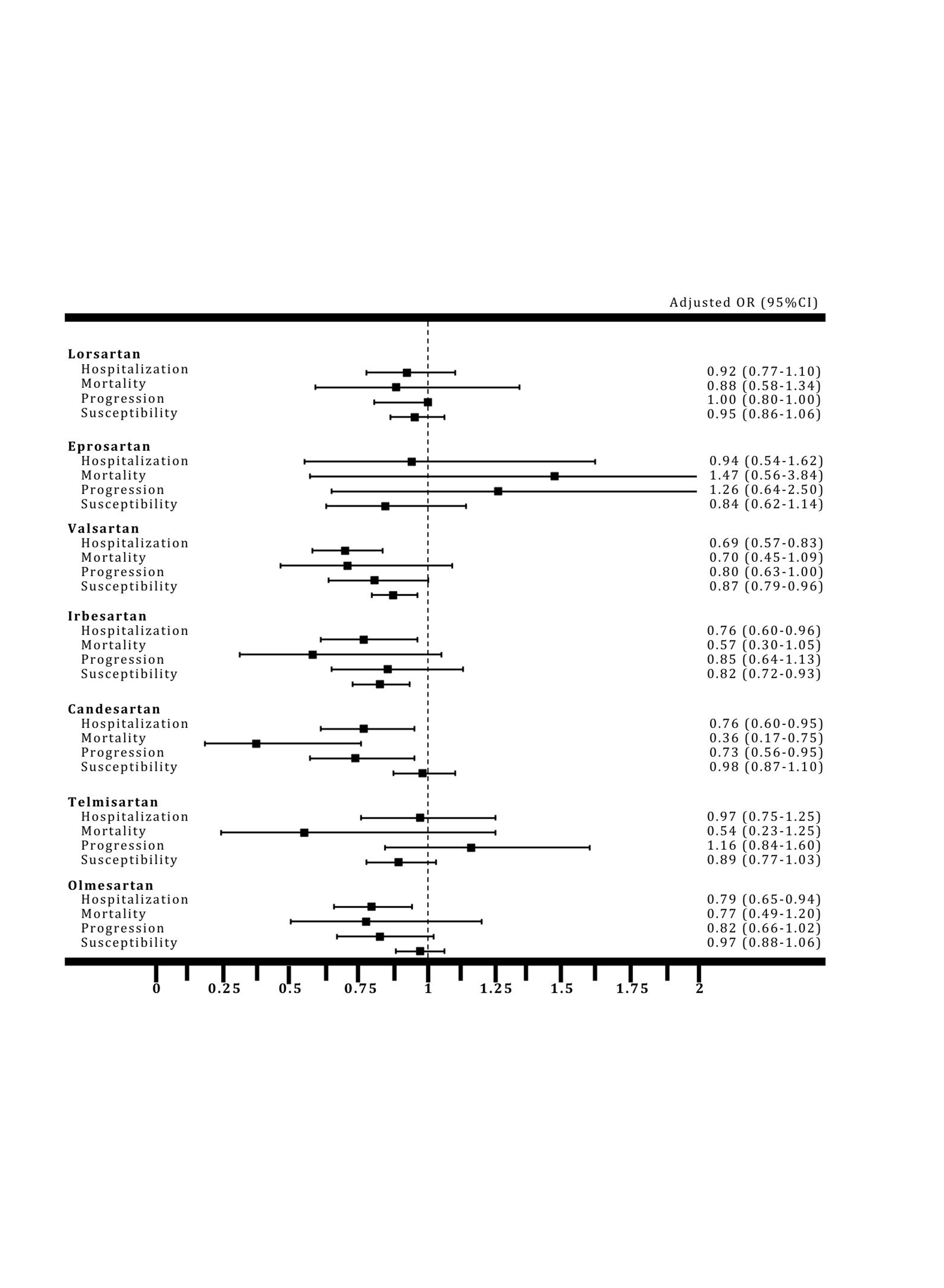

Supplement: Supplementary file 3 — Supplementary file3 (TIF 1575 KB) [file 10787_2024_1475_MOESM3_ESM.tif]

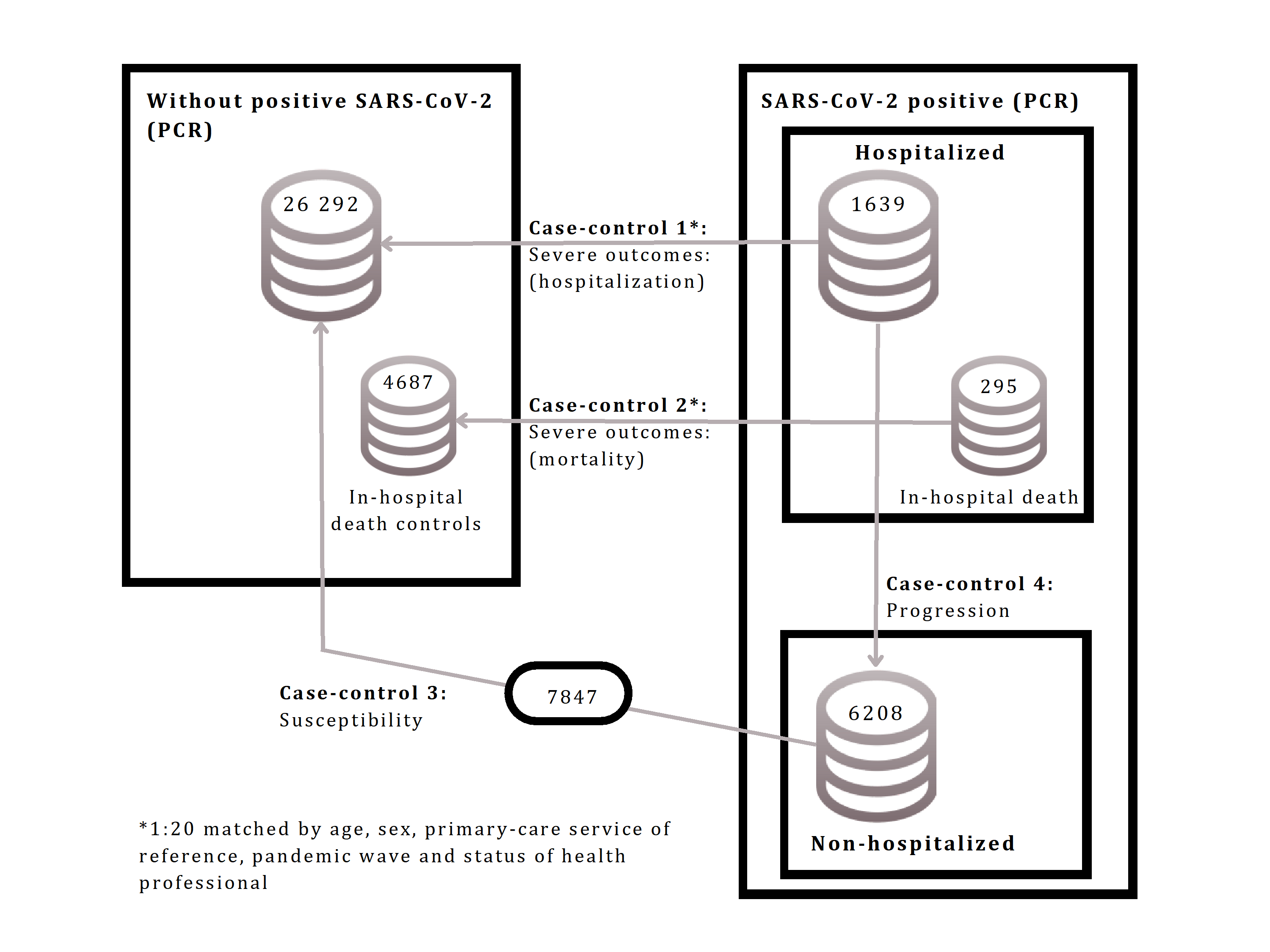

Supplement: Supplementary file 4 — Supplementary file4 (TIF 630 KB) [file 10787_2024_1475_MOESM4_ESM.tif]

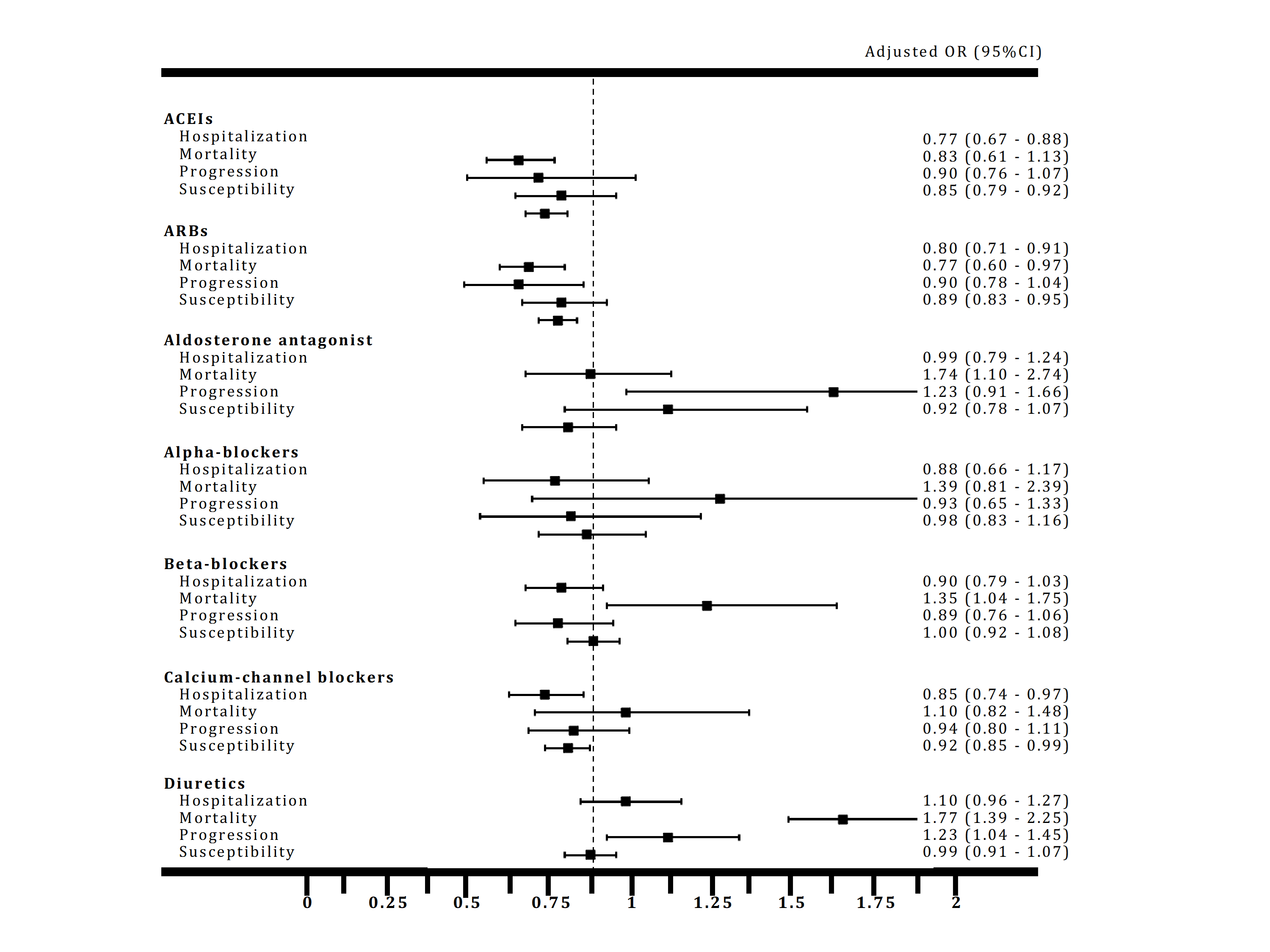

Supplement: Supplementary file 5 — Supplementary file5 (TIF 852 KB) [file 10787_2024_1475_MOESM5_ESM.tif]

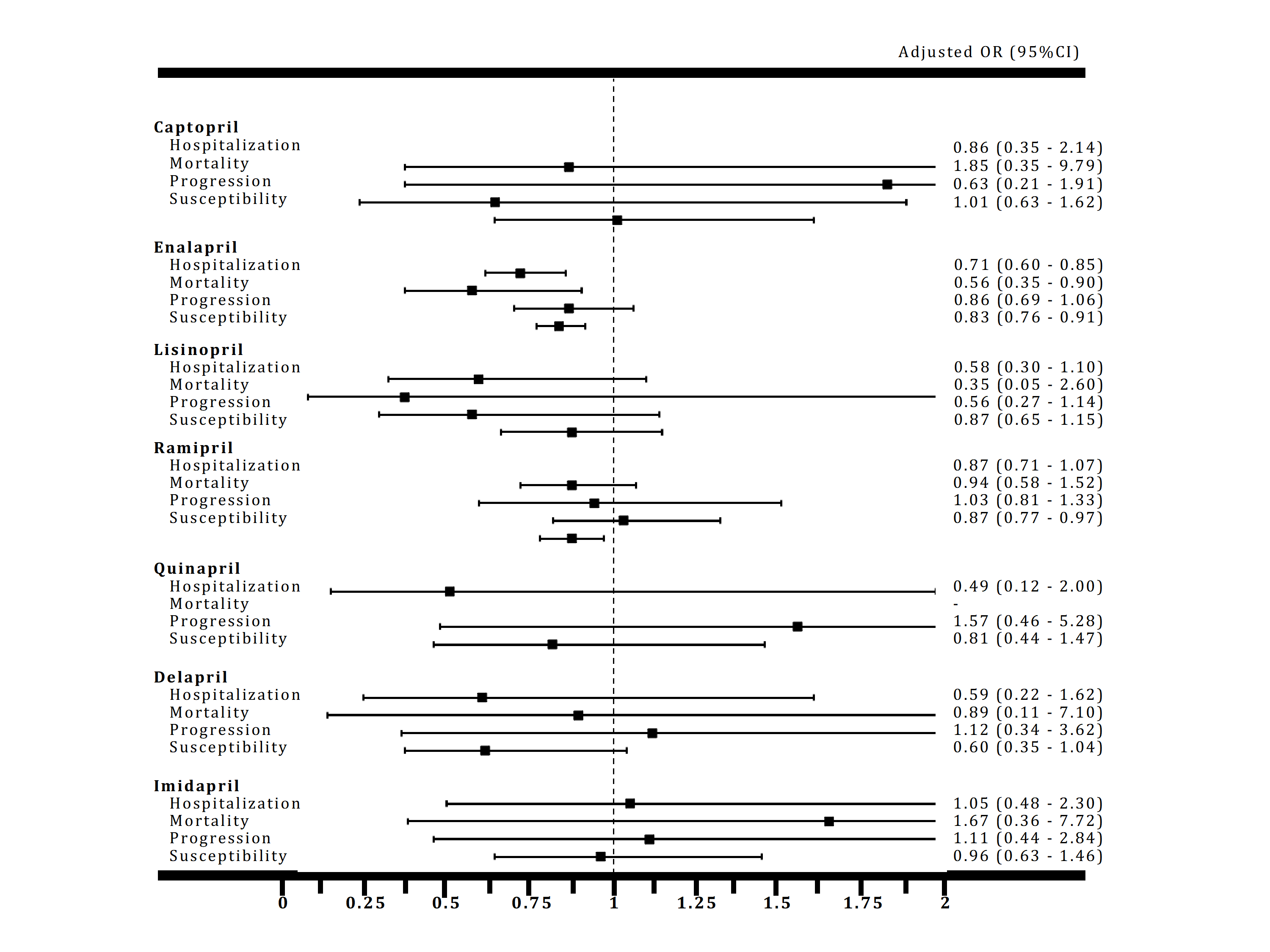

Supplement: Supplementary file 6 — Supplementary file6 (TIF 573 KB) [file 10787_2024_1475_MOESM6_ESM.tif]

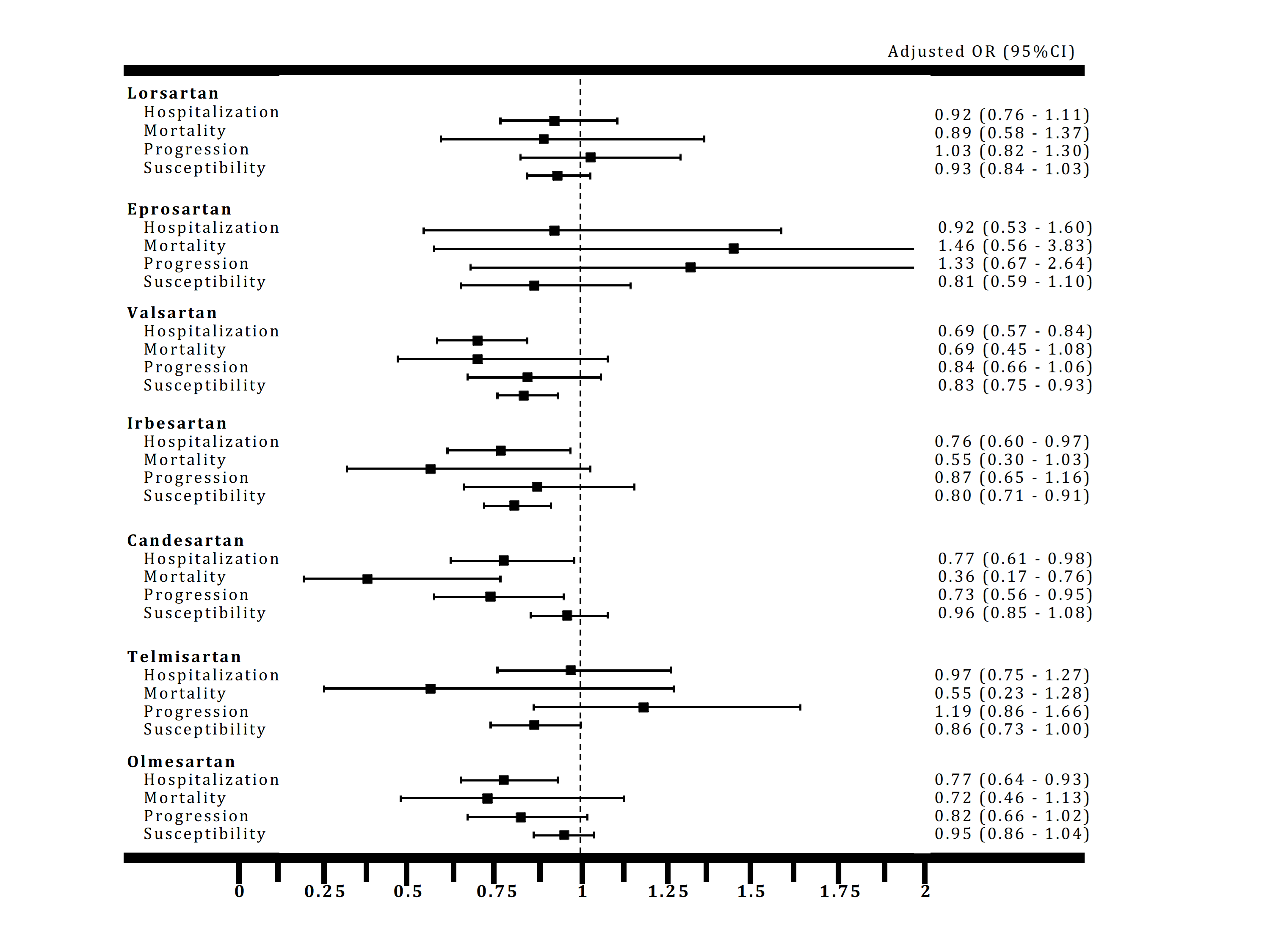

Supplement: Supplementary file 7 — Supplementary file7 (TIF 883 KB) [file 10787_2024_1475_MOESM7_ESM.tif]
