# Supplementary material for: Impact of prior antihypertensive treatment on COVID-19 outcomes, by active ingredient
Source: Inflammopharmacology. 2024 Apr 15;32(3):1805–15. doi: 10.1007/s10787-024-01475-2 (PMC11136854; doi:10.1007/s10787-024-01475-2)
Supplement: Supplementary file 8 — Supplementary file8 (DOCX 225 KB) [file 10787_2024_1475_MOESM8_ESM.docx]

***Impact of prior antihypertensive treatment on COVID-19 outcomes, by active ingredient***

[**Inflammopharmacology**](https://www.springer.com/journal/10787)

**Experimental and Therapeutic Studies**

- Rosa M García-Álvarez (1,2) ORCiD 0000-0003-4703-0234
- Maruxa Zapata-Cachafeiro (2,3,4) ORCiD: 0000-0002-0648-7716
- Irene Visos-Varela (2) ORCiD: 0000-0001-6466-621
- Almudena Rodríguez-Fernández (2,3,4) ORCiD 0000-0002-3350-1237
- Samuel Pintos-Rodríguez (2) ORCiD 0009-0007-8315-6463
- Maria Piñeiro-Lamas (4) ORCiD 0000-0003-3655-100X
- Teresa M Herdeiro (5) ORCiD 0000-0002-0500-4049
- Adolfo Figueiras (2,3,4) ORCiD 0000-0002-5766-8672
- Angel Salgado-Barreira (2,3,4) ORCiD 0000-0003-4349-4947
- COVIDrug Group: Rosendo Bugarín-González, Eduardo Carracedo-Martínez, Francisco J González-Barcala, Martina Lema-Oreiro, Narmeen Mallah, Manuel Portela-Romero, Angela Prieto-Campo, Marc Saez, Margarita Taracido-Trunk.

***Affiliations:***

1. Service of Preventive Medicine and Public Health, Clinic Hospital of Santiago de Compostela, Santiago de Compostela, Spain.
2. Department of Preventive Medicine and Public Health. University of Santiago de Compostela, Spain.
3. Institute of Health Research of Santiago de Compostela, Santiago de Compostela, Spain.
4. Consortium for Biomedical Research in Epidemiology & Public Health (CIBER en Epidemiología y Salud Pública-CIBERESP), University of Santiago de Compostela, Santiago de Compostela, Spain.
5. Department of Medical Sciences, iBiMED-Institute of Biomedicine, University of Aveiro, Aveiro, Portugal.

***Name and address for correspondence and reprint requests:***

Maruxa Zapata Cachafeiro

Department of Preventive Medicine and Public Health

C/ San Francisco s/n

University of Santiago de Compostela

15786 Santiago de Compostela

A Coruña (Spain)

e-mail: [maruxa.zapata@usc.es](mailto:maruxa.zapata@usc.es)

**Table S1** Summary of the 4 case-control substudies (global analysis)

| **Case-control** | **Aim** | **Cases** | **Controls** | **Matching** | **Index date** |
| --- | --- | --- | --- | --- | --- |
| - 1. **Severe COVID-19 outcomes - hospitalization** | To assess the effect of ACEIs/ARBs on risk of hospitalization for COVID-19. | All subjects >18 years admitted for COVID-19 (PCR+) in a GHS hospital (N=2821). | Subjects who did not present PCR+, matched with cases (N=52 318). | Yes* | Cases: 10 days prior to the PCR+  Controls: the same as for its matched case. |
| - 1. **Severe COVID-19 outcomes - mortality** | To assess the effect of ACEIs/ARBs on risk of mortality in patients with COVID-19. | All subjects >18 years hospitalized for COVID-19 who died during admission to any of the GHS hospitals in 2020 (N=397). | Subjects who did not present PCR+, matched with cases (N=7129). | Yes* | Cases: 10 days prior to the PCR+  Controls: the same as for its matched case. |
| 1. **Progression to severe COVID-19 outcomes** | To evaluate the effect of ACEIs/ARBs on disease progression to more severe stages that might require hospital admission. | Same cases as those in the Case-control 1 substudy (N=2821). | All patients with diagnosis of COVID-19 confirmed by PCR, who did not require hospitalization  (N=26 996). | No | Cases: 10 days prior to the PCR+  Controls: 10 days prior to the PCR+. |
| 1. **Susceptibility to the virus** | To establish the impact of ACEIs/ARBs on risk of infection. | All subjects over the age of 18 years with diagnosis of COVID-19, confirmed by PCR (both hospitalized and non-hospitalized)  (N=29 817). | Same controls as those in the Case-control 1 substudy  (N=52 318). | No | Cases: 10 days prior to the PCR+  Controls: the same as for cases of Case-control 1 substudy. |

GHS = Galician Health Service

*1:20 matched by age, sex, primary-care service of reference, pandemic wave and status of health professional

**Table S2** Antihypertensive drug subgroups by risk of severity (risk of hospitalization and mortality) (global analysis)

|  |  | **Severe COVID-19 outcomes** | | | | | | | | |
| --- | --- | --- | --- | --- | --- | --- | --- | --- | --- | --- |
|  | **Risk of hospitalization** | | | | |  | **Risk of mortality** | | | |
|  | CASES:  PCR+ hospitalized (N=2821) | | CONTROLS:  non-PCR+  (N=52 318) | Adjusted OR^a^  (95%CI) | P-value |  | CASES:  PCR+ deceased  (N=397) | CONTROLS:  non-PCR+  (N=7129) | Adjusted OR^a^  (95%CI) | P-value |
| ACEIs (C09AA) | 380 (13.5) | | 6856 (13.1) | 0.78 (0.69-0.89) | 0.004 |  | 61 (15.4) | 1120 (15.7) | 0.71 (0.52-0.98) | 0.039 |
| ARBs (C09CA) | 702 (24.9) | | 12 427 (23.8) | 0.80 (0.72-0.90) | <0.001 |  | 115 (29) | 2190 (30.7) | 0.69 (0.52-0.91) | 0.008 |
| Aldosterone antagonists (C03DA) | 136 (4.8) | | 1338 (2.6) | 1.03 (0.83-1.27) | 0.806 |  | 25 (6.3) | 254 (3.6) | 0.79 (0.49-1.29) | 0.352 |
| Alpha-blockers (C02CA) | 57 (2) | | 851 (1.6) | 0.85 (0.64-1.13) | 0.276 |  | 15 (3.8) | 183 (2.6) | 0.86 (0.48-1.57) | 0.631 |
| Beta-blockers (C07AB) | 486 (17.2) | | 7245 (13.8) | 0.96 (0.84-1.08) | 0.481 |  | 102 (25.7) | 1314 (18.4) | 1.04 (0.78-1.38) | 0.792 |
| Calcium-channel blockers (C08C) | 319 (11.3) | | 5255 (10) | 0.83 (0.73-0.95) | 0.006 |  | 62 (15.6) | 946 (13.3) | 0.78 (0.57-1.07) | 0.124 |
| Diuretics (C03) | 640 (22.7) | | 7832 (15) | 1.03 (0.91-1.17) | 0.622 |  | 144 (36.3) | 1565 (22) | 1.11 (0.83-1.48) | 0.469 |

OR = odds ratio; ARBs: angiotensin II receptor blockers; ACEIs: angiotensin converting enzyme inhibitors

^a^Adjusted for: sex, age, status of health professional, comorbidities (hypertension, diabetes, COPD, obesity, ischaemic heart disease, cerebrovascular accident, heart failure, atrial fibrillation, chronic renal failure, cancer, asthma, current smoker), current use of other pharmacological treatments and number of treatments for chronic diseases. Additionally, the primary-care service of reference and the pandemic wave were included as random effects

**Table S3** Antihypertensive drug subgroups by risk of severity (progression and susceptibility) (global analysis)

|  | **Progression to severe COVID-19 outcomes** | | | |  | **Susceptibility to the virus** | | | |
| --- | --- | --- | --- | --- | --- | --- | --- | --- | --- |
|  | CASES:  PCR+ cases  hospitalized  (N=2821) | CONTROLS: PCR+ non-hospitalized  (N=26 996) | Adjusted  OR^a^  (95%CI) | P-value |  | CASES: PCR+  hospitalized &  non-hospitalized  (N=29 817) | CONTROLS:  non-PCR+  (N=52318) | Adjusted  OR^a^  (95%CI) | P-value |
| ACEIs (C09AA) | 380 (13.5) | 6856 (13.1) | 0.90 (0.77-1.06) | 0.204 |  | 1893 (6.3) | 6856 (13.1) | 0.88 (0.82-0.94) | <0.001 |
| ARBs (C09CA) | 702 (24.9) | 12 427 (23.8) | 0.88 (0.76-1.00) | 0.057 |  | 3443 (11.5) | 12 427 (23.8) | 0.92 (0.86-0.97) | 0.005 |
| Aldosterone antagonists (C03DA) | 136 (4.8) | 1338 (2.6) | 1.24 (0.94-1.63) | 0.124 |  | 381 (1.3) | 1338 (2.6) | 0.94 (0.82-1.08) | 0.409 |
| Alpha-blockers (C02CA) | 57 (2) | 851 (1.6) | 0.93 (0.65-1.32) | 0.684 |  | 213 (0.7) | 851 (1.6) | 0.98 (0.83-1.16) | 0.848 |
| Beta-blockers (C07AB) | 486 (17.2) | 7245 (13.8) | 0.96 (0.83-1.11) | 0.596 |  | 2102 (7) | 7245 (13.8) | 1.01 (0.95-1.08) | 0.738 |
| Calcium-channel blockers (C08C) | 319 (11.3) | 5255 (10) | 0.89 (0.76-1.05) | 0.155 |  | 1362 (4.6) | 52255 (10) | 0.93 (0.87-1.00) | 0.053 |
| Diuretics (C03) | 640 (22.7) | 7832 (15) | 1.06 (0.91-1.24) | 0.432 |  | 2127 (7.1) | 7832 (15) | 1.02 (0.95-1.09) | 0.594 |

OR = odds ratio; ARBs: angiotensin II receptor blockers; ACEIs: angiotensin converting enzyme inhibitors

^a^Adjusted for: sex, age, status of health professional, comorbidities (hypertension, diabetes, COPD, obesity, ischaemic heart disease, cerebrovascular accident, heart failure, atrial fibrillation, chronic renal failure, cancer, asthma, current smoker), current use of other pharmacological treatments and number of treatments for chronic diseases. Additionally, the primary-care service of reference and the pandemic wave were included as random effects

**Table S4** Demographic and clinical characteristics of COVID-19 cases and matched controls (severe outcomes: hospitalization and mortality) (analysis by subgroups of hypertensive patients)

|  | **Severe COVID-19 outcomes** | | | | | |
| --- | --- | --- | --- | --- | --- | --- |
|  | **Hospitalization** | | |  | **Mortality** | |
| **Characteristic** | CASES:  PCR+ hospitalized (N=1639) | CONTROLS:  non-PCR+  (N=26 292) | |  | CASES:  PCR+ deceased  (N=295) | CONTROLS:  non-PCR+  (N=4687) |
| **Sex; n (%)** |  | |  |  |  |  |
| Male | 859(52.4) | 13 434 (51.1) | |  | 175 (59.3) | 2684 (57.3) |
| Female | 780 (47.6) | 12 858 (48.9) | |  | 120 (40.7) | 2003 (42.7) |
| **Age**, median (IQR) | 80 (70 - 87) | 80 (72 - 87) | |  | 85 (78 - 89) | 85 (78 - 88) |
| **Health professionals; n (%)** | 20 (1.2) | 246 (0.9) | |  | 0 (0.0) | 0 (0.0) |
| **Comorbidities; n (%)** |  | |  |  |  |  |
| Diabetes | 616 (37.6) | 7930 (30.2) | |  | 128 (43.4) | 1439 (30.7) |
| COPD | 272 (16.6) | 2980 (11.3) | |  | 69 (23.4) | 640 (13.7) |
| Obesity | 602 (36.7) | 7763 (29.5) | |  | 99 (33.6) | 1305 (27.8) |
| Ischaemic heart disease | 279 (17) | 3561 (13.5) | |  | 77 (26.1) | 740 (15.8) |
| Cerebrovascular accident | 220 (13.4) | 2860 (10.9) | |  | 54 (18.3) | 592 (12.6) |
| Heart failure | 374 (22.8) | 3209 (12.2) | |  | 96 (32.5) | 686 (14.6) |
| Atrial fibrillation | 355 (21.7) | 4416 (16.8) | |  | 68 (23.1) | 947 (20.2) |
| Chronic renal failure | 364 (22.2) | 3655 (13.9) | |  | 92 (31.2) | 807 (17.2) |
| Cancer | 334 (20.4) | 4713 (17.9) | |  | 70 (23.7) | 967 (20.6) |
| Asthma | 160 (9.8) | 1681 (6.4) | |  | 20 (6.8) | 264 (5.6) |
| Current smoker | 421 (25.7) | 3516 (13.4) | |  | 66 (22.4) | 555 (11.8) |

IQR= interquartile range; COPD = chronic obstructive pulmonary disease

**Table S5** Demographic and clinical characteristics of COVID-19 cases and matched controls (progression to severe COVID-19 outcomes and susceptibility to the virus) (analysis by subgroups of hypertensive patients)

|  | **Progression to severe COVID-19 outcomes** | | | | **Susceptibility to the virus** | | | |  |
| --- | --- | --- | --- | --- | --- | --- | --- | --- | --- |
| **Characteristic** | CASES:  PCR+ hospitalized (N=1639) | CONTROLS:  PCR+ non-hospitalized  (N=6208) | |  | | CASES: PCR+  hospitalized &  non-hospitalized  (N=7847) | | CONTROLS:  non-PCR+  (N=26 292) | |
| **Sex; n (%)** |  | |  | |  | |  | |  |
| Male | 859 (52.4) | 11 217 (41.6) | | | 3352 (42.7) | | 26 998 (51.6) | |  |
| Female | 780 (47.6) | 15 779 (58.4) | | | 4495 (57.3) | | 25320 (48.4) | |  |
| **Age**, median (IQR) | 80 (70 - 87) | 47 (33 – 63) | | | 74 (62 - 85) | | 73 (60 – 84) | |  |
| **Health professional; n (%)** | 20 (1.2) | 1238 (4.6) | | | 125 (1.6) | | 1203 (2.3) | |  |
| **Comorbidities; n (%)** |  | |  | |  | |  | |  |
| Diabetes | 616 (37.6) | 1716 (27.6) | | | 2332 (29.7) | | 7930 (30.2) | |  |
| COPD | 272 (16.6) | 473 (7.6) | | | 745 (9.5) | | 2980 (11.3) | |  |
| Obesity | 602 (36.7) | 1997 (32.2) | | | 2599 (33.1) | | 7763 (29.5) | |  |
| Ischaemic heart disease | 279 (17) | 628 (10.1) | | | 907 (11.6) | | 3561 (13.5) | |  |
| Cerebrovascular accident | 220 (13.4) | 626 (10.1) | | | 846 (10.8) | | 2860 (10.9) | |  |
| Heart failure | 374 (22.8) | 560 (9) | | | 934 (11.9) | | 3209 (12.2) | |  |
| Atrial fibrillation | 355 (21.7) | 837 (13.5) | | | 1192 (15.2) | | 4416 (16.8) | |  |
| Chronic renal failure | 364 (22.2) | 633 (10.2) | | | 997 (12.7) | | 3655 (13.9) | |  |
| Cancer | 334 (20.4) | 853 (13.7) | | | 1187 (15.1) | | 4713 (17.9) | |  |
| Asthma | 160 (9.8) | 472 (7.6) | | | 632 (8.1) | | 1681 (6.4) | |  |
| Current smoker | 421 (25.7) | 942 (15.2) | | | 1363 (17.4) | | 3516 (13.4) | |  |

IQR= interquartile range; COPD = Chronic obstructive pulmonary disease

**Table S6** Summary of the 4 case-control substudies (analysis by subgroups of hypertensive patients)

| **Case-control** | **Aim** | **Cases** | **Controls** | **Matching** | **Index date** |
| --- | --- | --- | --- | --- | --- |
| - 1. **Severe COVID-19 outcomes - hospitalization** | To assess the effect of ACEIs/ARBs on risk of hospitalization for COVID-19. | All subjects >18 years admitted for COVID-19 (PCR+) in a GHS hospital (N=1639). | Subjects who did not present PCR+, matched with cases (N=26 292). | Yes* | Cases: 10 days prior to the PCR+  Controls: the same as for its matched case. |
| - 1. **Severe COVID-19 outcomes - mortality** | To assess the effect of ACEIs/ARBs on risk of mortality in patients with COVID-19. | All subjects >18 years hospitalized for COVID-19 who died during admission to any of the GHS hospitals in 2020 (N=295). | Subjects who did not present PCR+, matched with cases (N=7129). | Yes* | Cases: 10 days prior to the PCR+  Controls: the same as for its matched case. |
| 1. **Progression to severe COVID-19 outcomes** | To evaluate the effect of ACEIs/ARBs on disease progression to more severe stages that might require hospital admission. | Same cases as those in the Case-control 1 substudy (N=1639). | All patients with diagnosis of COVID-19 confirmed by PCR, who did not require hospitalization  (N=6208). | No | Cases: 10 days prior to the PCR+  Controls: 10 days prior to the PCR+. |
| 1. **Susceptibility to the virus** | To establish the impact of ACEIs/ARBs on risk of infection. | All subjects over the age of 18 years with diagnosis of COVID-19, confirmed by PCR (both hospitalized and non-hospitalized)  (N=7847). | Same controls as those in the Case-control 1 substudy  (N=26 292). | No | Cases: 10 days prior to the PCR+  Controls: the same as for cases of Case-control 1 substudy. |

GHS = Galician Health Service

*1:20 matched by age, sex, primary-care service of reference, pandemic wave and status of health professional

**Table S7** Antihypertensive drug subgroups by risk of severity (risk of hospitalization and mortality) (analysis by subgroups of hypertensive patients)

|  |  | **Severe COVID-19 outcomes** | | | | | | | | |
| --- | --- | --- | --- | --- | --- | --- | --- | --- | --- | --- |
|  | **Risk of hospitalization** | | | | |  | **Risk of mortality** | | | |
|  | CASES:  PCR+ hospitalized (N=1639) | | CONTROLS:  non-PCR+  (N=26 292) | Adjusted OR^a^  (95%CI) | P-value |  | CASES:  PCR+ deceased  (N=295) | CONTROLS:  non-PCR+  (N=4687) | Adjusted OR^a^  (95%CI) | P-value |
| ACEIs (C09AA) | 347 (21.2) | | 6055 (23) | 0.77 (0.67 - 0.88) | <0.001 |  | 53 (18) | 978 (20.9) | 0.83 (0.61 - 1.13) | 0.024 |
| ARBs (C09CA) | 692 (42.2) | | 11978 (45.6) | 0.80 (0.71 - 0.91) | <0.001 |  | 114 (38.6) | 2116 (45.1) | 0.77 (0.60 - 0.97) | 0.007 |
| Aldosterone antagonists (C03DA) | 117 (7.1) | | 1107 (4.2) | 0.99 (0.79 - 1.24) | 0.941 |  | 22 (7.5) | 208 (4.4) | 1.74 (1.1 - 2.74) | 0.476 |
| Alpha-blockers (C02CA) | 56 (3.4) | | 808 (3.1) | 0.88 (0.66 - 1.17) | 0.366 |  | 15 (5.1) | 174 (3.7) | 1.39 (0.81 - 2.39) | 0.817 |
| Beta-blockers (C07AB) | 401 (24.5) | | 5814 (22.1) | 0.90 (0.79 - 1.03) | 0.141 |  | 85 (28.8) | 1084 (23.1) | 1.35 (1.04 - 1.75) | 0.838 |
| Calcium-channel blockers (C08C) | 309 (18.9) | | 4884 (18.6) | 0.85 (0.74 - 0.97) | 0.017 |  | 60 (20.3) | 880 (18.8) | 1.10 (0.82 - 1.48) | 0.208 |
| Diuretics (C03) | 562 (34.3) | | 6656 (25.3) | 1.10 (0.96 - 1.27) | 0.163 |  | 122 (41.4) | 1336 (28.5) | 1.77 (1.39 - 2.25) | 0.940 |

OR = odds ratio; ARBs: angiotensin II receptor blockers; ACEIs: angiotensin converting enzyme inhibitors

^a^Adjusted for: sex, age, status of health professional, comorbidities (diabetes, COPD, obesity, ischaemic heart disease, cerebrovascular accident, heart failure, atrial fibrillation, chronic renal failure, cancer, asthma, current smoker), current use of other pharmacological treatments and number of treatments for chronic diseases. Additionally, the primary-care service of reference and the pandemic wave were included as random effects

**Table S8** Antihypertensive drug subgroups by risk of severity (progression and susceptibility) (analysis by subgroups of hypertensive patients)

|  | **Progression to severe COVID-19 outcomes** | | | |  | **Susceptibility to the virus** | | | |
| --- | --- | --- | --- | --- | --- | --- | --- | --- | --- |
|  | CASES:  PCR+ cases  hospitalized  (N=1639) | CONTROLS: PCR+ non-hospitalized  (N=6208) | Adjusted  OR^a^  (95%CI) | P-value |  | CASES: PCR+  hospitalized &  non-hospitalized  (N=7847) | CONTROLS:  non-PCR+  (N=26 292) | Adjusted  OR^a^  (95%CI) | P-value |
| ACEIs (C09AA) | 347 (21.2) | 1311 (21.1) | 0.90 (0.76 - 1.07) | 0.229 |  | 1658 (21.1) | 6055 (23) | 0.85 (0.79 - 0.92) | <0.001 |
| ARBs (C09CA) | 692 (42.2) | 2607 (42) | 0.90 (0.78 - 1.04) | 0.165 |  | 3299 (42) | 11978 (45.6) | 0.89 (0.83 - 0.95) | <0.001 |
| Aldosterone antagonists (C03DA) | 117 (7.1) | 179 (2.9) | 1.23 (0.91 - 1.66) | 0.176 |  | 296 (3.8) | 1107 (4.2) | 0.92 (0.78 - 1.07) | 0.847 |
| Alpha-blockers (C02CA) | 56 (3.4) | 146 (2.4) | 0.93 (0.65 - 1.33) | 0.676 |  | 202 (2.6) | 808 (3.1) | 0.98 (0.83 - 1.16) | 0.847 |
| Beta-blockers (C07AB) | 401 (24.5) | 1165 (18.8) | 0.89 (0.76 - 1.06) | 0.185 |  | 1566 (20) | 5814 (22.1) | 1.00 (0.92 - 1.08) | 0.914 |
| Calcium-channel blockers (C08C) | 309 (18.9) | 955 (15.4) | 0.94 (0.8 - 1.11) | 0.493 |  | 1264 (16.1) | 4884 (18.6) | 0.92 (0.85 - 0.99) | 0.028 |
| Diuretics (C03) | 562 (34.3) | 1168 (18.8) | 1.23 (1.04 - 1.45) | 0.017 |  | 1730 (22) | 6656 (25.3) | 0.99 (0.91 - 1.07) | 0.740 |

OR = odds ratio; ARBs: angiotensin II receptor blockers; ACEIs: angiotensin converting enzyme inhibitors

^a^Adjusted for: sex, age, status of health professional, comorbidities (diabetes, COPD, obesity, ischaemic heart disease, cerebrovascular accident, heart failure, atrial fibrillation, chronic renal failure, cancer, asthma, current smoker), current use of other pharmacological treatments and number of treatments for chronic diseases. Additionally, the primary-care service of reference and the pandemic wave were included as random effects

**Table S9** Severe COVID-19 outcomes (ACEI): risk of hospitalization and mortality (analysis by subgroups of hypertensive patients)

|  | **Severe COVID-19 outcomes** | | | | | | | | |
| --- | --- | --- | --- | --- | --- | --- | --- | --- | --- |
|  | **Risk of hospitalization** | | | | | **Risk of mortality** | | | |
|  | CASES:  PCR+ hospitalized (N=1639) | CONTROLS:  non-PCR+  (N=26 292) | Adjusted  OR^a^  (95%CI) | P-value |  | CASES:  PCR+ deceased  (N=295) | CONTROLS:  non-PCR+  (N=4687) | Adjusted  OR^a^  (95%CI) | P-value |
| **ACEIs (C09AA)** | 347 (21.2)^b^ | 6055 (23)^b^ | 0.77 (0.67 - 0.88) | <0.001 |  | 53 (18)^b^ | 978 (20.9)^b^ | 0.67 (0.47 - 0.95) | 0.024 |
| Captopril (C09AA01) | 5 (0.3) | 95 (0.4) | 0.86 (0.35 - 2.14) | 0.743 |  | 2 (0.7) | 10 (0.2) | 1.85 (0.35 - 9.79) | 0.469 |
| Enalapril (C09AA02) | 171 (10.4) | 3352 (12.7) | 0.71 (0.60 - 0.85) | <0.001 |  | 22 (7.5) | 537 (11.5) | 0.56 (0.35 - 0.90) | 0.016 |
| Lisinopril (C09AA03) | 10 (0.6) | 237 (0.9) | 0.58 (0.30 - 1.10) | 0.095 |  | 1 (0.3) | 35 (0.7) | 0.35 (0.05 - 2.60) | 0.303 |
| Ramipril (C09AA05) | 125 (7.6) | 1744 (6.6) | 0.87 (0.71 - 1.07) | 0.179 |  | 23 (7.8) | 289 (6.2) | 0.94 (0.58 - 1.52) | 0.789 |
| Quinapril (C09AA06) | 2 (0.1) | 71 (0.3) | 0.49 (0.12 - 2.00) | 0.318 |  | 0 (0) | 10 (0.2) | - | - |
| Fosinopril (C09AA09) | 1 (0.1) | 27 (0.1) | 0.65 (0.09 - 4.82) | 0.671 |  | 1 (0.3) | 5 (0.1) | 2.96 (0.33 - 26.57) | 0.333 |
| Delapril (C09AA12) | 4 (0.2) | 103 (0.4) | 0.59 (0.22 - 1.62) | 0.309 |  | 1 (0.3) | 15 (0.3) | 0.89 (0.11 - 7.10) | 0.915 |
| Imidapril (C09AA16) | 7 (0.4) | 101 (0.4) | 1.05 (0.48 - 2.30) | 0.893 |  | 2 (0.7) | 17 (0.4) | 1.67 (0.36 - 7.72) | 0.513 |

OR = odds ratio; ACEIs= angiotensin converting enzyme inhibitors

^a^Adjusted for: sex, age, status of health professional, comorbidities (diabetes, COPD, obesity, ischaemic heart disease, cerebrovascular accident, heart failure, atrial fibrillation, chronic renal failure, cancer, asthma, current smoker), current use of other pharmacological treatments and number of treatments for chronic diseases. Additionally, the primary-care service of reference and the pandemic wave were included as random effects

^b^The overall number of subjects exposed to ACEIs (C09AA) is lower than the sum of those exposed to the active ingredients of individual ACEIs (C09AA01, C09AA02, C09AA03, C09AA05, C09AA06, C09AA09, C09AA12, C09AA16), due to the fact that some subjects were exposed to more than one ACEI across the study period

**Table S10** Progression to severe COVID-19 outcomes and susceptibility to the virus (ACEI) (analysis by subgroups of hypertensive patients)

|  | **Progression to severe COVID-19 outcomes** | | | | | **Susceptibility to the virus** | | | |
| --- | --- | --- | --- | --- | --- | --- | --- | --- | --- |
|  | CASES:  PCR+ cases  hospitalized  (N=1639) | CONTROLS:  PCR+ non-hospitalized  (N=6208) | Adjusted  OR^a^  (95%CI) | P-value |  | CASES: PCR+  hospitalized &  non-hospitalized  (N=7847) | CONTROLS:  non-PCR+  (N=26 292) | Adjusted  OR^a^  (95%CI) | P-value |
| **ACEIs (C09AA)** | 347 (21.2)^b^ | 1311 (21.1)^b^ | 0.90 (0.76 - 1.07) | 0.229 |  | 1658 (21.1)^b^ | 6055 (23)^b^ | 0.85 (0.79 - 0.92) | <0.001 |
| Captopril (C09AA01) | 5 (0.3) | 20 (0.3) | 0.63 (0.21 - 1.91) | 0.413 |  | 25 (0.3) | 95 (0.4) | 1.01 (0.63 - 1.62) | 0.972 |
| Enalapril (C09AA02) | 171 (10.4) | 723 (11.6) | 0.86 (0.69 - 1.06) | 0.155 |  | 894 (11.4) | 3352 (12.7) | 0.83 (0.76 - 0.91) | <0.001 |
| Lisinopril (C09AA03) | 10 (0.6) | 65 (1) | 0.56 (0.27 - 1.14) | 0.110 |  | 75 (1) | 237 (0.9) | 0.87 (0.65 - 1.15) | 0.327 |
| Ramipril (C09AA05) | 125 (7.6) | 370 (6) | 1.03 (0.81 - 1.33) | 0.788 |  | 495 (6.3) | 1744 (6.6) | 0.87 (0.77 - 0.97) | 0.017 |
| Quinapril (C09AA06) | 2 (0.1) | 13 (0.2) | 1.57 (0.46-5.28) | 0.833 |  | 15 (0.2) | 71 (0.3) | 0.81 (0.44 - 1.47) | 0.482 |
| Fosinopril (C09AA09) | 1 (0.1) | 4 (0.1) | 0.81 (0.08 - 7.85) | 0.854 |  | 5 (0.1) | 27 (0.1) | 0.76 (0.28 - 2.06) | 0.591 |
| Delapril (C09AA12) | 4 (0.2) | 13 (0.2) | 1.12 (0.34 - 3.62) | 0.853 |  | 17 (0.2) | 103 (0.4) | 0.60 (0.35 - 1.04) | 0.068 |
| Imidapril (C09AA16) | 7 (0.4) | 28 (0.5) | 1.11 (0.44 - 2.84) | 0.824 |  | 35 (0.4) | 101 (0.4) | 0.96 (0.63 - 1.46) | 0.860 |

OR = odds ratio; ACEIs= angiotensin converting enzyme inhibitors

^a^Adjusted for: sex, age, status of health professional, comorbidities (diabetes, COPD, obesity, ischaemic heart disease, cerebrovascular accident, heart failure, atrial fibrillation, chronic renal failure, cancer, asthma, current smoker), current use of other pharmacological treatments and number of treatments for chronic diseases. Additionally, the primary-care service of reference and the pandemic wave were included as random effects

^b^The overall number of subjects exposed to ACEIs (C09AA) is lower than the sum of those exposed to the active ingredients of individual ACEIs (C09AA01, C09AA02, C09AA03, C09AA05, C09AA06, C09AA09, C09AA12, C09AA16), due to the fact that some subjects were exposed to more than one ACEI across the study period

**Table S11** Severe COVID-19 outcomes (ARBs): risk of hospitalization and mortality (analysis by subgroups of hypertensive patients)

|  | **Severe COVID-19 outcomes** | | | | | | | | |
| --- | --- | --- | --- | --- | --- | --- | --- | --- | --- |
|  | **Risk of hospitalization** | | | | | **Risk of mortality** | | | |
|  | CASES:  PCR+ hospitalized (N=1639) | CONTROLS:  non-PCR+  (N=26 292) | Adjusted  OR^a^  (95%CI) | P-value |  | CASES:  PCR+ deceased  (N=295) | CONTROLS:  non-PCR+  (N=4687) | Adjusted  OR^a^  (95%CI) | P-value |
| **ARBs (C09CA)** | 692 (42.2)^b^ | 11978 (45.6)^b^ | 0.77 (0.67 - 0.88) | <0.001 | | 114 (38.6)^b^ | 2116 (45.1)^b^ | 0.68 (0.51 - 0.90) | 0.008 |
| Losartan (C09CA01) | 154 (9.4) | 2209 (8.4) | 0.92 (0.76 - 1.11) | 0.387 | | 31 (10.5) | 408 (8.7) | 0.89 (0.58 - 1.37) | 0.610 |
| Eprosartan (C09CA02) | 14 (0.9) | 250 (1) | 0.92 (0.53 - 1.60) | 0.777 | | 5 (1.7) | 50 (1.1) | 1.46 (0.56 - 3.83) | 0.441 |
| Valsartan (C09CA03) | 135 (8.2) | 2611 (9.9) | 0.69 (0.57 - 0.84) | <0.001 | | 28 (9.5) | 475 (10.1) | 0.69 (0.45 - 1.08) | 0.104 |
| Irbesartan (C09CA04) | 83 (2.9) | 1649 (3.2) | 0.76 (0.60 - 0.97) | 0.028 | | 12 (4.1) | 279 (6) | 0.55 (0.30 - 1.03) | 0.064 |
| Candesartan (C09CA06) | 88 (5.4) | 1592 (6.1) | 0.77 (0.61 - 0.98) | 0.032 | | 8 (2.7) | 287 (6.1) | 0.36 (0.17 - 0.76) | 0.007 |
| Telmisartan (C09CA07) | 69 (4.2) | 1037 (3.9) | 0.97 (0.75 - 1.27) | 0.836 | | 6 (2) | 174 (3.7) | 0.55 (0.23 - 1.28) | 0.162 |
| Olmesartan (C09CA08) | 159 (9.7) | 2865 (10.9) | 0.77 (0.64 - 0.93) | 0.006 | | 28 (9.5) | 482 (10.3) | 0.72 (0.46 - 1.13) | 0.154 |

OR = odds ratio; ARBs: angiotensin II receptor blockers

^a^Adjusted for: sex, age, status of health professional, comorbidities (diabetes, COPD, obesity, ischaemic heart disease, cerebrovascular accident, heart failure, atrial fibrillation, chronic renal failure, cancer, asthma, current smoker), current use of other pharmacological treatments and number of treatments for chronic diseases. Additionally, the primary-care service of reference and the pandemic wave were included as random effects

^b^The overall number of subjects exposed to ARBs (C09CA) is lower than the sum of those exposed to the active ingredients of individual ARBs (C09CA01, C09CA02, C09CA03, C09CA04, C09CA06, C09CA07, C09CA08), due to the fact that some subjects were exposed to more than one ARB across the study period

**Table S12** Progression to severe COVID-19 outcomes and susceptibility to the virus (ARBs) (analysis by subgroups of hypertensive patients)

|  | **Progression to severe COVID-19 outcomes** | | | | | **Susceptibility to the virus** | | | |
| --- | --- | --- | --- | --- | --- | --- | --- | --- | --- |
|  | CASES:  PCR+ cases  hospitalized  (N=1639) | CONTROLS: PCR+ non-hospitalized  (N=6208) | Adjusted  OR^a^  (95%CI) | P-value |  | CASES: PCR+  hospitalized &  non-hospitalized  (N=7847) | CONTROLS:  non-PCR+  (N=26 292) | Adjusted  OR^a^  (95%CI) | P-value |
| **ARBs (C09CA)** | 692 (42.2)^b^ | 2607 (42)^b^ | 0.90 (0.78 - 1.04) | 0.165 | | 3299 (42)^b^ | 11978 (45.6) | 0.89 (0.83 - 0.95) | <0.001 |
| Losartan (C09CA01) | 154 (9.4) | 501 (8.1) | 1.03 (0.82 - 1.30) | 0.785 | | 655 (8.3) | 2209 (8.4) | 0.93 (0.84 - 1.03) | 0.164 |
| Eprosartan (C09CA02) | 14 (0.9) | 41 (0.7) | 1.33 (0.67 - 2.64) | 0.416 | | 55 (0.7) | 250 (1) | 0.81 (0.59 - 1.10) | 0.180 |
| Valsartan (C09CA03) | 135 (8.2) | 528 (8.5) | 0.84 (0.66 - 1.06) | 0.136 | | 663 (8.4) | 2611 (9.9) | 0.83 (0.75 - 0.93) | <0.001 |
| Irbesartan (C09CA04) | 83 (5.1) | 312 (5) | 0.87 (0.65 - 1.16) | 0.326 | | 395 (5) | 1587 (6) | 0.80 (0.71 - 0.91) | <0.001 |
| Candesartan (C09CA06) | 88 (5.4) | 371 (6) | 0.73 (0.56-0.95) | 0.057 | | 459 (5.8) | 1592 (6.1) | 0.96 (0.85 - 1.08) | 0.472 |
| Telmisartan (C09CA07) | 69 (4.2) | 185 (3) | 1.19 (0.86 - 1.66) | 0.286 | | 254 (3.2) | 1037 (3.9) | 0.86 (0.73 - 1.00) | 0.048 |
| Olmesartan (C09CA08) | 159 (9.7) | 704 (11.3) | 0.82 (0.66-1.02) | 0.077 | | 863 (11) | 2865 (10.9) | 0.95 (0.86 - 1.04) | 0.259 |

OR = odds ratio; ARBs: angiotensin II receptor blockers

^a^Adjusted for: sex, age, status of health professional, comorbidities (diabetes, COPD, obesity, ischaemic heart disease, cerebrovascular accident, heart failure, atrial fibrillation, chronic renal failure, cancer, asthma, current smoker), current use of other pharmacological treatments and number of treatments for chronic diseases. Additionally, the primary-care service of reference and the pandemic wave were included as random effects

^b^The overall number of subjects exposed to ARBs (C09CA) is lower than the sum of those exposed to the active ingredients of individual ARBs (C09CA01, C09CA02, C09CA03, C09CA04, C09CA06, C09CA07, C09CA08), due to the fact that some subjects were exposed to more than one ARB across the study period
